# Development of an LDL Receptor-Targeted Peptide Susceptible to Facilitate the Brain Access of Diagnostic or Therapeutic Agents

**DOI:** 10.3390/biology9070161

**Published:** 2020-07-11

**Authors:** Séverine André, Lionel Larbanoix, Sébastien Verteneuil, Dimitri Stanicki, Denis Nonclercq, Luce Vander Elst, Sophie Laurent, Robert N. Muller, Carmen Burtea

**Affiliations:** 1 NMR and Molecular Imaging Laboratory, Department of General, Organic and Biomedical Chemistry, University of Mons, Avenue Maistriau 19, Mendeleïev Building, B-7000 Mons, Belgium; Severine.ANDRE@umons.ac.be (S.A.); Sebastien.Verteneuil@uliege.be (S.V.); Dimitri.STANICKI@umons.ac.be (D.S.); Luce.VANDERELST@umons.ac.be (L.V.E.); Sophie.LAURENT@umons.ac.be (S.L.); Robert.MULLER@umons.ac.be (R.N.M.); 2 Center for Microscopy and Molecular Imaging, rue Adrienne Bolland 8, B-6041 Gosselies, Belgium; Lionel.LARBANOIX@umons.ac.be; 3 Department of Histology, University of Mons, Pentagon—1B, Avenue du Champ de Mars 6, B-7000 Mons, Belgium; Denis.NONCLERCQ@umons.ac.be

**Keywords:** LDL receptor, brain delivery, peptides, phage display, ultrasmall superparamagnetic particles of iron oxide, CF770

## Abstract

Blood-brain barrier (BBB) crossing and brain penetration are really challenging for the delivery of therapeutic agents and imaging probes. The development of new crossing strategies is needed, and a wide range of approaches (invasive or not) have been proposed so far. The receptor-mediated transcytosis is an attractive mechanism, allowing the non-invasive penetration of the BBB. Among available targets, the low-density lipoprotein (LDL) receptor (LDLR) shows favorable characteristics mainly because of the lysosome-bypassed pathway of LDL delivery to the brain, allowing an intact discharge of the carried ligand to the brain targets. The phage display technology was employed to identify a dodecapeptide targeted to the extracellular domain of LDLR (ED-LDLR). This peptide was able to bind the ED-LDLR in the presence of natural ligands and dissociated at acidic pH and in the absence of calcium, in a similar manner as the LDL. In vitro, our peptide was endocytosed by endothelial cells through the caveolae-dependent pathway, proper to the LDLR route in BBB, suggesting the prevention of its lysosomal degradation. The in vivo studies performed by magnetic resonance imaging and fluorescent lifetime imaging suggested the brain penetration of this ED-LDLR-targeted peptide.

## 1. Introduction

The blood-brain barrier (BBB) is a structure at the interface between the brain and the blood that strictly controls the brain homeostasis in association with the blood-cerebrospinal fluid barrier (BCSFB) and the ependymal barrier [1]. BBB is composed of a monolayer of endothelial cells (EC) joined by tight junctions, limiting the paracellular crossing, and surrounded by astrocytes and pericytes. In addition to the brain’s physical protection, BBB exhibits biological characteristics improving its efficacy, such as a well-developed enzymatic function, a low number of pinocytosis vesicles, and a high proportion of mitochondria, reflecting its important metabolic activity [2]. Due to the presence of the BBB, most drugs are not able to passively access the brain if they do not meet certain characteristics, such as lipophilicity and a size smaller than 400 Daltons [3].

The development of new BBB crossing strategies is a real challenge, and some invasive and non-invasive methods are available [2,4]. The first group (i.e., ultrasounds, microwaves, osmotic opening, etc.) leads to the BBB disruption (transient or not), which precludes their routine clinical implementation due to the crucial role played by this barrier in brain protection and homeostasis. The second group shows more interest by employing natural pathways to allow brain access while the BBB’s integrity is preserved. If the use of the nasal pathway [5] and of non-specific pathways, such as the passive diffusion (lipidization) or the adsorptive-mediated transcytosis (cationization), is possible [6], the receptor-mediated transcytosis (RMT) offers the advantage to be specific. It involves the binding of a vector (i.e., endogenous ligand, antibody, or peptide), coupled with the molecule of interest, to a receptor that initiates the endocytosis of this receptor and leads to the transcytosis of the complex across the EC [7,8]. The most studied receptors used for this purpose are the transferrin receptor (TfR), the insulin receptor (IR), and the low-density lipoprotein receptor (LDLR) and its related proteins (LRP1 and LRP2).

The LDLR is a ubiquitous transmembrane receptor of a large family of receptors involved in lipid metabolism. It recognizes a variety of ligands, among which apolipoproteins B (apoB) and E (apoE) are present in lipoprotein particles. The binding of these particles leads to their endocytosis through clathrin-coated pits and their transfer to the lysosome degradation pathway that delivers cholesterol for cellular exploitation [9]. On the other hand, the caveolae-mediated endocytosis seems to be followed by the LDLR to cross over the BBB, whereas the lysosomal degradation is shown to be bypassed [10,11]. Moreover, although brain cholesterol is mainly produced *in situ*, LDLR has been shown to be preferentially expressed at the apical membrane of the brain EC, suggesting its involvement in an endocytosis mechanism in these cells [12]. Finally, even if brain RNA databases reveal lower LDLR levels than TfR or IR [13,14,15,16], the targeting of LDLR shows advantages compared to them. For instance, the high blood concentration of Tf potentially prevents the binding of synthetic ligands to the TfR [17], whereas IR targeting shows adverse effects, such as hypoglycemia [7]. All these characteristics make the LDLR an attractive target for BBB crossing.

In order to identify a new vector that could improve brain access of therapeutic or diagnostic molecules, a randomized library of phage-displayed linear dodecapeptides was screened on the extracellular domain of the LDLR (ED-LDLR). The phage display technology is a powerful method for peptide screening, allowing the identification of specific peptides against a target [18]. Peptides show interesting advantages compared to larger molecules, such as lower toxicity and immunogenicity [19]. However, they present short half-life and poor bioavailability and stability, which can be improved by their molecular optimization [20].

The peptide LRPep2 (LDL receptor-peptide 2), described in this work, was selected based on interesting characteristics during the phage display experiments, mostly in terms of affinity and competitive binding with natural ligands. It was then evaluated in vitro with the aim to understand its mechanism of endocytosis in brain EC. Aiming to explore its potential to penetrate the mouse brain in vivo, LRPep2 was coupled to ultrasmall superparamagnetic particles of iron oxide (USPIO-LRPep2) and detected by magnetic resonance imaging (MRI). The biodistribution of USPIO-LRPep2 was studied by nuclear magnetic resonance (NMR) relaxometry and histology after mice euthanasia. Finally, the brain penetration of LDLR-targeted peptide was furthermore assessed by fluorescent lifetime imaging (FLI) after coupling LRPep2 to the fluorescent dye CF770 (CF770-LRPep2).

## 2. Materials and Methods

### 2.1. Phage Display Experiments

A phage-displayed random library of linear dodecapeptides (Ph.D.-12, New England Biolabs Inc., Bioké, Leiden, The Netherlands) was screened against the ED-LDLR (Recombinant Human LDLR, R&D Systems, Abingdon, Oxon, UK), as previously described [21]. The selection of the hits was based on (a) the apparent dissociation constants (K*_d_); (b) the half-maximal inhibitory concentration (IC_50_) of natural ligands; (c) the influence of pH and calcium on ED-LDLR binding. These evaluations were specific to the target. Complete protocols are available in Supplementary Materials_Methods.

### 2.2. Docking of the Selected Peptides to ED-LDLR

The interaction of the selected peptides LRPep1 (LDL receptor-peptide 1) and LRPep2 (LDL receptor-peptide 2) with ED-LDLR has been studied using the HPEPDOCK web server (http://huanglab.phys.hust.edu.cn/hpepdock/) [22]. This program employs a docking algorithm, which considers that linear peptides can adopt a wide range of spatial conformations. Among the generated docking models, 10 of them are proposed as the top binding prediction models. The crystallographic structure of ED-LDLR can be either uploaded as a PDB file or is provided by the server after introducing the sequence in a FASTA format or the PDB ID of the protein, i.e., 3M0C chain C for the sequence 4-788 of LDLR. The quality of docking is evaluated based on the root-mean-square deviation (RMSD) that considers the atoms of the peptide and protein residues located within 10 Å of distance. A successful docking prediction is indicated by an RMSD ≤ 2.0 Å.

### 2.3. Cell Culture

ACBRI376 cells (primary human brain microvascular EC) were cultured in complete Complete Classic Medium (Cell Systems, Kirkland, WA, USA) supplemented with 1% antibiotic-antimycotic (Fisher Scientific, Brussels, Belgium) and 2% CultureBoost (Cell Systems). According to the manufacturer, these cells issued from human brain cortex tissue express after plating Cluster of Differentiation 31 (CD31) and von Willebrand Factor, known as EC markers, as well as Zonula Occludens 1 (ZO-1), a biomarker of tight junctions.

HepaRG (hepatocyte cell line) was maintained in William’s E medium supplemented with 10% fetal bovine serum (FBS), 13% thaw, plate, and general purpose medium supplement, and 1% GlutaMAX (all from Fisher Scientific). N18(H) (neuroblastoma cell line) and 1321N1 (astrocytoma cell line) cells were cultured in DMEM (4.5 g/L glucose, L-glutamine, sodium pyruvate) supplemented with 10% FBS and penicillin/streptomycin 1% for N18(H) or 2% for 1321N1 (all from Fisher Scientific). HUVEC (human umbilical vein EC) cells were cultured in MCDB131 medium supplemented with 20% FBS, 1% L-glutamine, 1% antibiotic-antimycotic, and 0.14% heparin 5000 U/mL (all from Fisher Scientific).

Experiments on N18(H) cells were performed after differentiation. Cells were immobilized on the appropriate support, and the differentiation was induced the second day, using medium containing 0.2% FBS for 48 h. 

### 2.4. Evaluation of the Endocytosis Potential of Peptides LRPep1 and LRPep2

Cells were seeded onto coverslips coated with collagen (0.2 mg/mL, Sigma-Aldrich, Overijse, Belgium) at a density of 8 × 10^5^ cells/well and grown for 3 days. At this time, cells did not form a monolayer in order to properly distinguish them and observe peptides’ endocytosis.

Cells were incubated for 2 h at 37 °C in the dark with LRPep1-rho or LRPep2-rho (peptides coupled to rhodamine, 200 µM in culture medium for HepaRG and HUVEC, 25 µM for ACBRI376 and 1321N1); the negative control was incubated with culture medium. The use of the rhodamine as a fluorescent probe was based on our previous experience with labeled peptides [23]. Cells were rinsed two times with PBS (per liter: 8 g NaCl, 0.2 g KCl, 2.31 g Na_2_HPO_4_ × 12 H_2_O, 0.2 g KH_2_PO_4_, pH 7.4). The Hoechst solution (Hoechst 33342 trihydrochloride, Fisher Scientific) prepared at 2 µg/mL in HBSS (per liter: 0.140 g CaCl_2_, 0.1 g MgCl_2_ × 6H_2_O, 0.4 g KCl, 0.06 g KH_2_PO_4_, 0.35 g NaHCO_3_, 8 g NaCl, 0.121 g Na_2_HPO_4_ × 12H_2_O, pH 7.4) was incubated for 5 min to stain nuclei. Cells were rinsed two times and mounted with HBSS. 

Fluorescence was observed using a Leica DM2000 microscope equipped with a light source EL 6000 and a DFC 425C camera (Leica Microsystems, Groot Bijgaarden, Belgium).

### 2.5. Evaluation of the LDLR Expression in Cells and Colocalization of LRPep2 with LDLR

Cells seeded onto coverslips, as previously described, were rinsed two times with PBS and fixed using 4% buffered paraformaldehyde for 15 min. Then, the cells were permeabilized with methanol 100% for 10 min at −20 °C. Between each step, cells were rinsed two times with PBS. Finally, cells were blocked with PBS supplemented with 5% normal goat serum (NGS, Cell Signaling Technology, Leiden, The Netherlands) and 0.3% Triton X-100 (Sigma-Aldrich). LDLR was detected using the anti-LDLR antibody made in rabbit (# PA5-22976, recognizing residues 500–550 of human and mouse LDLR that present a 92% identity, and both glycosylated (~150kDa) and non-glycosylated LDLR (95 kDa); Thermo Fisher Scientific, Erembodegem, Belgium), incubated overnight at 7 µg/mL in PBS and the anti-rabbit IgG made in goat coupled to Texas Red (Vector Labconsult, Brussels, Belgium) incubated 1 h at 20 µg/mL in phosphate buffer (Na_2_HPO_4_ × 12 H_2_O 10 mM, NaH_2_PO_4_ × H_2_O 10 mM, NaCl 150 mM, pH 7.8) supplemented with 0.5% bovine serum albumin (BSA). After a final rinsing step, they were mounted using Vectashield mounting medium with 4′,6-diamidine-2′-phenylindole dihydrochloride (DAPI, Vector Labconsult).

For the colocalization of LRPep2-rho with LDLR (7 µg/mL of anti-LDLR antibody made in rabbit) on EC, cells were fixed only with methanol, and the peptide LRPep2-rho was incubated at 10 µM during the incubation with the secondary antibody (20 µg/mL of anti-rabbit IgG antibody made in goat and coupled to fluorescein, Vector Labconsult).

### 2.6. Evaluation of LDLR Expression on Mouse Brain Slices and Colocalization of LRPep2 with LDLR

Slices (5 µm thickness) were obtained from the brain of healthy NMRI mice (Naval Medical Research Institute, Harlan, Horst, The Netherlands), fixed in 4% paraformaldehyde solution (Sigma-Aldrich, Bornem, Belgium) and paraffin-embedded. Slices were rehydrated, and antigen retrieval was performed using citrate buffer (C_6_H_5_Na_3_O_7_ ∙ 2H_2_O 10 mM, Tween 20 0.05%, pH 6.0). Slices were then rinsed with PBS (3 × 5 min).

For LDLR detection by immunohistochemistry (IHC), slices were successively blocked for 15 min with H_2_O_2_ 0.7% prepared in PBS, streptavidin, and biotin (both from Vector Labconsult, incubation at 37 °C). Slices were rinsed between each step with PBS supplemented with Tween-20 0.1% (2 × 5 min). They were finally blocked with protein-free (TBS) blocking buffer (PFBB, Pierce, Fisher Scientific) for one hour, rinsed in PBS 0.1% Tween-20 and PBS before overnight incubation at 4 °C with the anti-LDLR antibody made in rabbit prepared at 10 µg/mL in PBS. Slices were rinsed 3 times in PBS-0.1% Tween-20, then incubated for one hour with a biotinylated anti-rabbit IgG made in goat (20 µg/mL, Vector Labconsult) prepared in phosphate buffer. Slices were rinsed two times in PBS, incubated with the Vectastain ABC kit (Vector Labconsult) for one hour, and rinsed again. After 5 min with Tris-HCl 50 mM, the revelation was performed using a solution of 0.05% 3,3′-Diaminobenzidine (DAB) tetrachlorhydrate (Sigma-Aldrich) supplemented with 0.02% H_2_O_2_ prepared in PBS, pH 7.4. After staining, slices were rinsed, counterstained using Mayer’s Hemalun (VWR International, Leuven, Belgium) and Luxol Fast Blue, and mounted in a permanent medium (Leica Microsystems). Images were acquired using the Leica DM2000 microscope equipped with a DFC 425C camera.

For colocalization of LRPep2 with LDLR by immunofluorescence (IF), slices were blocked for one hour with PBS supplemented with 1% BSA. After rinsing in PBS-0.1% Tween-20 and PBS, the LDLR was detected using the antibody anti-LDLR made in the rabbit prepared at 3.5 µg/mL in PBS and incubated overnight at 4 °C. Slices were rinsed 3 times in PBS-0.1% Tween-20. Then, the peptide LRPep2-rho (10 µM) and the anti-rabbit IgG made in goat and coupled to fluorescein (5 µg/mL, Vector Labconsult) were prepared in phosphate buffer supplemented with 0.05% BSA and 0.5% Tween-20 and incubated with slices for 2 h. After rinsing again with PBS-0.1% Tween-20, slices were mounted using Vectashield mounting medium with DAPI.

### 2.7. Colocalization of the Peptide LRPep2 with Caveolae and Lysosomes

Cells were first incubated with LRPep2-rho, as described above (cf. 2.4. Evaluation of the endocytosis potential of peptides LRPep1 and LRPep2), before being permeabilized with methanol. Then, cells were blocked with PBS supplemented with 5% NGS and 0.3% Triton X-100. Caveolae and lysosomes were detected using anti-caveolin 1 and anti-LAMP1 (Lysosomal-Associated Membrane Protein 1) antibodies, respectively, made in rabbit (both from Santa Cruz, Heidelberg, Germany) and incubated overnight at 4 µg/mL in PBS. Finally, cells were incubated for 1 h with an anti-rabbit IgG made in goat coupled to fluorescein (Vector Labconsult) at 20 µg/mL in phosphate buffer pH 7.8 supplemented with 0.5% BSA. Cells were mounted with Vectashield mounting medium with DAPI.

### 2.8. Synthesis of USPIO Derivatives

The peptide LRPep2 or the peptide NSP (non-specific peptide: HSCNKNSCT, a scramble of a VCAM-1 (Vascular Cell Adhesion Molecule 1) binding peptide [24]; both synthesized by Eurogentec, Seraing, Belgium), presenting a molecule of polyethylene glycol (PEG, 8-amino-3,6-dioxaoctanoyl) at their N-terminus, was covalently grafted to the carboxylic groups of the USPIO, as previously described [25,26,27]. Then, a coating of PEG [O-(2-aminoethyl)-O-methyl-polyethyleneglycol, MW~750 g/mol, Sigma-Aldrich] was added in order to saturate free carboxyl groups. Due to the high concentration of peptides used for grafting, the non-conjugated peptides in USPIO suspensions were removed by extensive dialysis (MWCO: 30 kDa, Millipore, Burlington, MA, USA), whereas the concentration of peptides coupled to USPIO was determined using the Coomassie (Bradford) protein assay kit (Thermo Fisher Scientific). The absorbance of USPIO-PEG, corresponding to non-grafted USPIO, was subtracted from those of USPIO-LRPep2 and USPIO-NSP in order to remove the contribution of USPIO themselves (approximatively 62% of the total signal). Based on this measurement, it has been estimated that 1–2 peptides are bound per particle by considering that each particle contains ~11,000 Fe atoms [28].

### 2.9. Evaluation of the Affinity of USPIO-LRPep2 by ELISA

The K*_d_ of USPIO-LRPep2 was evaluated using a protocol similar to that used for phage clones and described in Supplementary Materials_Methods. Briefly, after the target immobilization, wells were blocked with PFBB and incubated with a range of 12 dilutions 1:1 of USPIO-LRPep2 starting at 7.36 × 10^−6^ M in TBSC (Tris-buffered saline containing calcium: Tris-HCl 50 mM, NaCl 150 mM, CaCl_2_ 2 mM, pH 7.4). Rinsing buffer was TBSC supplemented with 0.05% Tween-20 (TBSC-T). USPIO-LRPep2 was detected using a rabbit anti-PEG antibody at 2 µg/mL (Abcam) in TBSC-T supplemented with 0.5% BSA, a biotinylated anti-rabbit IgG made in goat at 5 µg/mL in phosphate buffer pH 7.8, and Vectastain ABC kit (both from Vector Labconsult).

### 2.10. MRI Experiments and Contrast Analysis Measurement

All in vivo experiments are in accordance with UMONS Animal Care and Use Committee (protocol MU-10-01 for the period July 2010–July 2014 and MU-10-02 for the period September 2014–September 2019). The mean number of animals used for in vivo studies was calculated using the power and sample size calculation software [29] based on the analysis of different vectorized nanoparticles investigated in vivo by our group [23,30,31,32,33].

Molecular imaging by MRI was performed on 6 NMRI mice (RjHan:NMRI, Janvier laboratories, St Berthevin, France) anesthetized with Nembutal 50 mg/kg body weight (b.w.; Sanofi, Brussels, Belgium) during MRI acquisitions. A small-animal monitoring and gating system was used to monitor the animal respiration rate, and the body temperature was maintained at 37 °C.

MRI images were acquired at the level of the head with T_2_-weighted RARE (rapid acquisition with relaxation enhancement) imaging protocol (TR/TE [repetition time/echo time] = 3000/60 ms, RARE factor = 4, NEX [number of excitations] = 4, matrix = 512 × 512, FOV [field-of-view] = 2.5 cm, slice thickness 1 mm, 20 axial slices, spatial resolution = 48 µm, TA [acquisition time] = 25 min 36 s) on a 300 MHz (7T) Bruker Pharmascan imaging system (Bruker, Ettlingen, Germany) equipped with a horizontal magnet and a circular polarized MRI transceiver coil (55 mm × 23 mm, frequency of 3 MHz, maximum RF [radiofrequency] of 5 ms). After pre-contrast acquisitions, USPIO derivatives were injected in the tail vein (3 mice/USPIO derivative) at a dose of 200 µmol Fe/kg b.w and a follow-up until approximatively 4 h post-injection was performed as well as an acquisition at 22 h.

The contrast was analyzed using the ImageJ software (National Institute of Health, Bethesda, MD, USA). The whole brain was selected manually, and the signal intensity (SI) of this region of interest (ROI) was measured on pre- (SI_pre_) and post-contrast (SI_post_) images. The standard deviation (SD) of the noise was measured in a region outside of the animal’s head. The percentage change of signal-to-noise ratio (Δ%SNR) on post-contrast images was calculated as follows:

(1) $$\Delta \%\mathrm{SNR}=\left[\frac{\left({\mathrm{SI}}_{\mathrm{post}}/\mathrm{Noise}\;\mathrm{SD})-({\mathrm{SI}}_{\mathrm{pre}}/\mathrm{Noise}\;\mathrm{SD}\right)}{\left({\mathrm{SI}}_{\mathrm{pre}}/\mathrm{Noise}\;\mathrm{SD}\right)}\right]\times 100$$

### 2.11. Organ Collection for Immunohistochemistry or Biodistribution Studies

NMRI mice were injected with USPIO derivatives or with PBS (*n* = 3/experimental group) and euthanized 55 min later by an injection of a lethal dose of Nembutal (500 mg/kg b.w) corresponding to the optimal timing of contrast enhancement determined by MRI. The blood and the urine were harvested, and the circulatory system was rinsed by transcardial perfusion of 5 mL of PBS injected two times in the left ventricle. Then, the brains, as well as the kidneys, the liver, and the spleen, were also collected for IHC and biodistribution studies. For IHC, brains were fixed by immersion in 4% paraformaldehyde for 24 h, followed by dehydration in successive baths of alcohol and butanol and paraffin embedding. Plasma was isolated by centrifugation at 7000 rpm for 30 min. The organs, plasma, and urines were conserved at −20 °C before using them in biodistribution studies.

### 2.12. Evaluation of the Biodistribution of UPSIO Derivatives by NMR Relaxometry

Organs, plasma, and urine conserved at −20 °C were placed in pyrex tubes for NMR analysis. The transversal relaxation time of water protons (T_2_) of each sample was measured on a Minispec Mq60 analyzer (60 MHz, 37 °C, Bruker, Karlsruhe, Germany). The relaxation rate of each organ (R_2_ = 1/T_2_) was calculated and normalized by the subtraction of the mean R_2_ of control mice (R_2_^Norm^ = R_2_^Sample^ − R_2_^Control^). For plasma and urine, the normalized R_2_ of each sample was related to the r_2_ (relaxivity) obtained for each USPIO derivative at 1 mM in plasma or urine controls, allowing to calculate the concentration of USPIO derivatives in plasma and urines of injected mice. 

The passage of USPIO derivatives in brains was also evaluated by the dosage of the iron contained in USPIO. Brains were recovered from pyrex tubes and dried in Eppendorf at 65 °C for 48 h. The weight of each sample was measured before digestion in 2 mL of HNO_3_-H_2_O_2_ (ratio 3:1) by microwaves (2 cycles: 5 min 250 W, 5 min 400 W, 5 min 650 W, 5 min 250 W; Milestone MSL-1200, Sorisole, Italy). The longitudinal relaxation time (T_1_) of each sample was measured on the Minispec Mq60 analyzer, the R_1_ was calculated (R_1_ = 1/T_1_), and the R_1_ of the blank (digestion solution) was subtracted. The iron concentration was obtained using a standard curve of iron. Concentrations were normalized to the final volume of digestion and to the dry weight of each sample. Mean iron content in brains of non-injected control mice was finally subtracted from that of mice injected with USPIO derivatives.

### 2.13. Detection of USPIO Derivatives on Mouse Brains by Perls’-DAB Staining of Iron

Brain slices (5 µm thickness) from injected mice were dewaxed, rehydrated, and blocked with 1% H_2_O_2_ in PBS for 15 min. After the rinsing steps (3 × 5 min in distilled water), slices were incubated in Perls’ solution (5% potassium ferrocyanide and 5% HCl in equal proportions) for 30 min. After rinsing 3 times (10 min) in distilled water, a solution of 0.05% DAB was added for 10 min. Finally, the revelation was performed using 0.05% DAB supplemented with 0.033% H_2_O_2_ prepared in PBS, pH 7.4. After staining, slices were rinsed, counterstained using Mayer’s Hemalun and Luxol Fast Blue, and mounted in a permanent medium. Images were acquired using the Leica DM2000 microscope equipped with a DFC 425C camera.

### 2.14. FLI Experiments and Fluorescence Measurement

Molecular imaging by FLI was performed on 9 nude mice (NU(NCr)-Foxn1<nu>, Charles River Laboratories, L’Arbresle, France). Mice were anesthetized with 4% isoflurane in O_2_ at a rate of 2 L/min, then maintained with 2% isoflurane at 0.3 L/min. For these experiments, our LDLR-targeted peptide was coupled to the fluorescent dye CF770 (CF770-LRPep2, Biosynthesis, Lewisville, TX, USA), whereas the fluorescent dye alone (VWR International) was used as the control.

FLI images were acquired with the PhotonIMAGER Optima (Ex = 737 nm, Em = 797 nm, TA = 5 s; BioSpace Lab, Nesles la Vallée, France). After pre-injection acquisitions, CF770-LRPep2 (*n* = 6 mice) and CF770 (*n* = 3 mice) were injected in the tail vein at a dose of 800 nmol per kg b.w., and the images were acquired at 25 min and 50 min post-injection. Mice were finally euthanized, and brains were collected (*n* = 3 for each compound) for ex vivo acquisitions (lens = 65 mm with f = 2.8, distance to lens = 279 mm).

The fluorescence analysis was performed using the M3Vision software (BioSpace Lab). On whole mouse images, the brain area was selected, and the fluorescent signal of this ROI was measured on pre- and post-injection images in photons by second, square centimeter, and steradian (ph/s/cm^2^/sr). The ratio “signal post-iv/signal pre-iv” was calculated for both compounds. On ex vivo images, an ROI was drawn in order to select the entire signal emitted by the brain, including the signal “outside” the brains. This signal was normalized to the signal emitted by the brain of a non-injected mouse.

### 2.15. Statistical Analysis

The results are expressed as means ± standard deviation (SD). The statistical analysis between experimental groups was performed using one-way ANOVA with SigmaPlot 11.0 software when data showed a normal distribution. For the non-normal distribution of the data, the Mann–Whitney test (non-parametric test) was used.

## 3. Results and Discussion

### 3.1. Selection of the LDLR-Targeted Peptides

**Figure 1:**
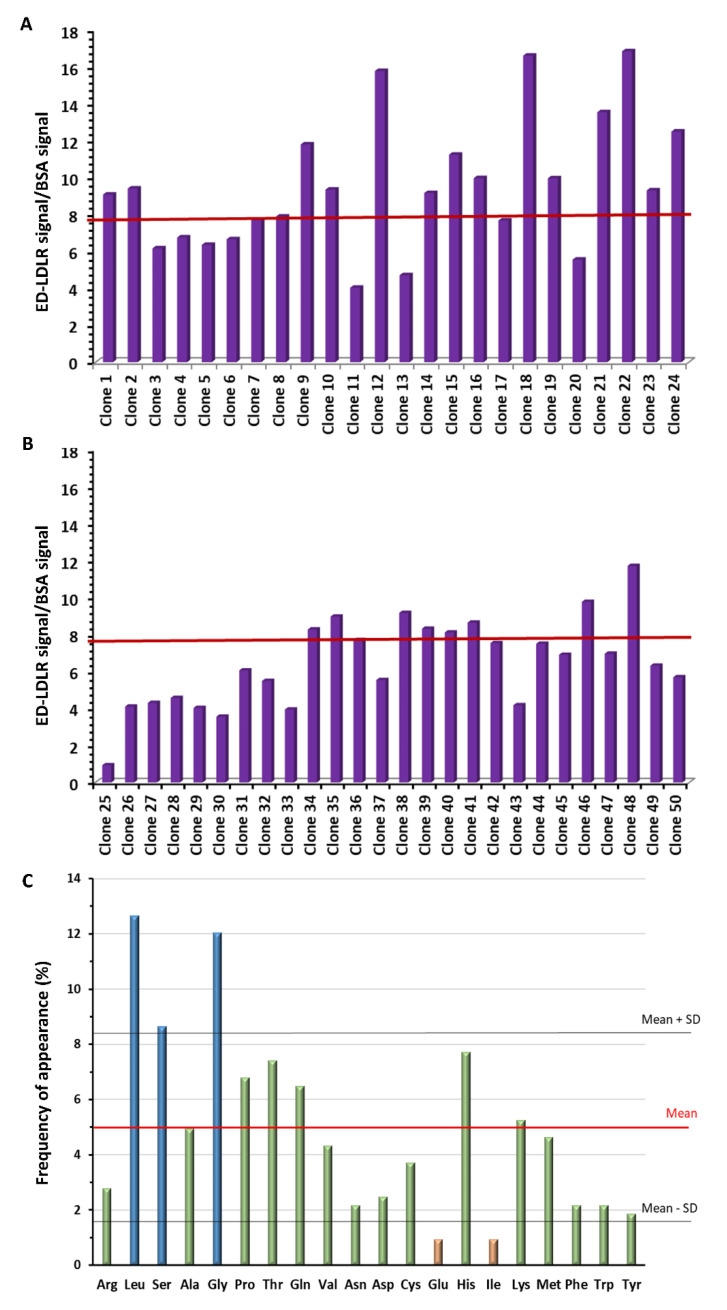
(**A**,**B**) Specific binding of the 50 clones isolated from the pool of the 3rd round of panning, determined by the ratio between the binding to the extracellular domain of low-density lipoprotein receptor (ED-LDLR) relative to bovine serum albumin (BSA) (see Figure S1). (**A**) Clones 1 to 24. (**B**) Clones 25 to 50. (**C**) Frequency of amino acids in the 13 different peptide sequences obtained after DNA sequencing.

The human LDLR is a transmembrane protein composed of 839 amino acids (Ala^22^–Ala^860^), whose N-terminal region spans almost the entire molecule (Ala^22^–Arg^788^) and is extracellular. The seven Cys-rich type A repeats (R1–R7) of the extracellular domain are responsible for ligand binding, i.e., ApoB100 and ApoE-comprising lipoprotein particles [9,34]. In our work, a randomized library of linear dodecapeptides expressed at the N-terminus of pIII minor coat protein of M13 bacteriophage was screened against the ED-LDLR (Ala^22^–Arg^788^). Three rounds of selection were performed to obtain a pool of phages with an increasing affinity to the target. Fifty clones were isolated from this 3rd pool of phages, and their binding to the ED-LDLR and the BSA employed as a control protein was evaluated at one concentration (Figure S1). The ratio between signals obtained against them allowed us to determine their specific binding to the target (Figure 1A,B). Among them, 29 clones presenting a specific binding ≥ to the mean (7.93 ± 3.32) were selected as hit candidates. Their DNA was isolated and sequenced, revealing 13 different peptide sequences (Table 1). Seven peptides (expressed by 18 clones) had probability to be expressed (P) of >90%, meaning that their selection might have been promoted by their high representation in the library.

The analysis of these peptide sequences showed different consensus motifs (GH, PT, QGGQ, KV) as well as Cys pairs present in four sequences, probably participating in the tridimensional conformation of peptides through disulfide bridge constrains. It is known that LDLR, ApoE, and ApoB present intramolecular disulfide bonds, which are crucial for molecule stabilization and ligand binding (LDLR) [34], or for dimerization (ApoE) [35], assembly, and secretion of hepatic lipoproteins (ApoB) [36]. Moreover, the analysis of amino acid frequencies (Figure 1C) revealed that three amino acids were more frequent (L, S, and G), with a percentage above the mean ± SD. Leu and Gly are important in the tertiary conformation of proteins; the side chain of Leu being relatively rigid, whereas Gly allows high flexibility [37].

**Figure 2:**
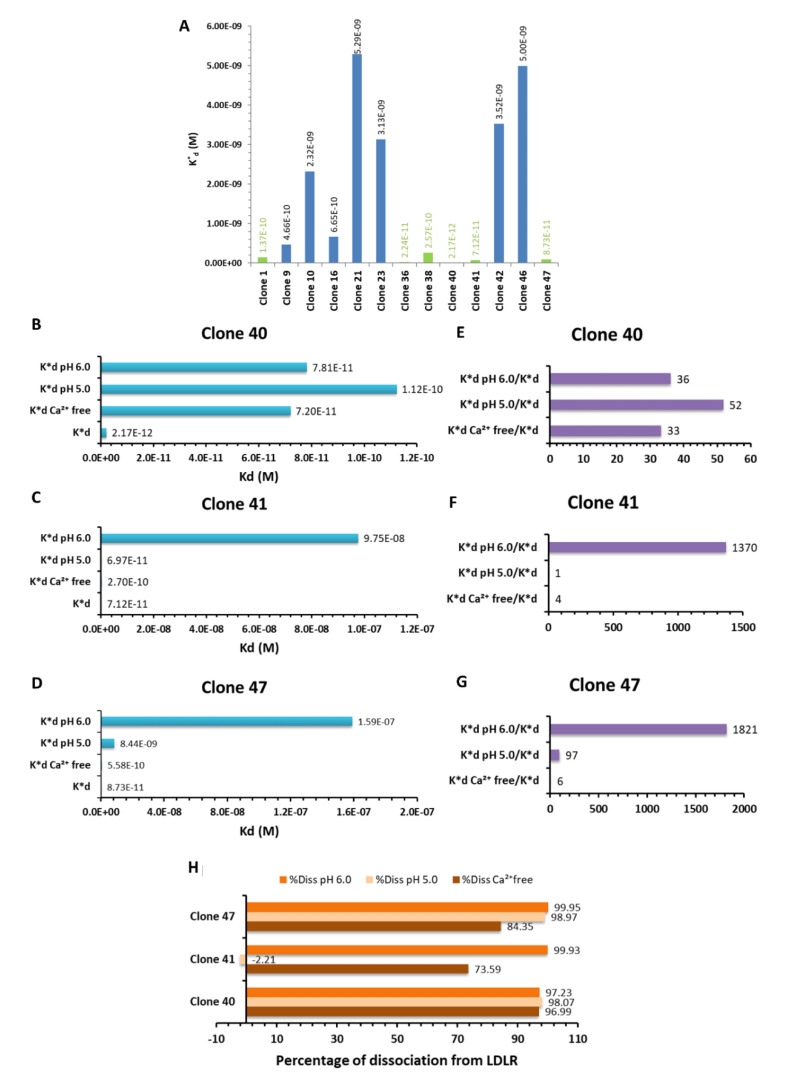
Binding of selected clones to ED-LDLR in various experimental conditions. (**A**) Apparent dissociation constants (K*_d_) of the 13 representative clones selected from the phage display experiments. Clones with low K*_d_, revealing high affinities and selected for further characterization, are in green. (**B**–**D**) K*_d_ of the clones 40, 41, and 47 in normal conditions, at acidic pH (pH 6.0, pH 5.0), and in the absence of calcium (Ca^2+^ free). (**E**–**G**) Ratios between K*_d_ in modified conditions and K*_d_ of the clones, reflecting the inhibitory effects of these modifications on the clones binding. (**H**) Percentage of dissociation of the clones from the LDLR.

The K*_d_ of the clones expressing these 13 sequences (one clone per sequence) were evaluated (Figure 2A). Based on these results, six clones (clones 1, 36, 38, 40, 41, 47, highlighted in green in Figure 2A) were selected for further characterizations, their K*_d_ being in the order of 10^−10^–10^−12^ M.

The following selection of the hits was based on their ability to bind the target in the presence of the natural ligands of LDLR, ApoB, and ApoE, respectively. The IC_50_ of ApoB and ApoE reflected the concentration of competitor required to block 50% of the clone’s binding. The IC_50_ value was thus directly proportional to the strength of the clone’s binding to ED-LDLR (described by its K*_d_), meaning that a high concentration of competitor was needed to destabilize the clone from its binding site. The ratios IC_50_/K*_d_ (Table 2) described the efficacy of the phage clone binding to the ED-LDLR in the presence of a competitor, being directly proportional to IC_50_ and inversely proportional to K*_d_. In other terms, the higher the IC_50_/K*_d_ ratio, the stronger was the phage clone binding to ED-LDLR. Clones 1 and 36 were highly destabilized by competitors, whereas the clones 38, 40, and 47 seemed to be more stable, e.g., the binding of the clone 40 was inhibited at 50% in the presence of ApoE at a concentration 1537-fold higher than the K*_d_ of this clone. Clones 40 and 47 were thus selected because of the highest ratios. Furthermore, we decided to select the clone 41 because of its lower K*_d_ than the clone 38, even if ratios IC_50_/K*_d_ showed that this clone might be destabilized by ApoB and ApoE.

The final selection was performed by the evaluation of the clones’ behavior in the absence of calcium and at acidic pH. Indeed, the calcium is necessary for the binding of ApoB and ApoE to the receptor [34,38], while a modification of pH induces different conformations of the receptor, an acidic pH in endosomes being responsible for the dissociation of ligands from the LDLR [34].

The K*_d_ of clones 40, 41, and 47 at acidic pH and in the absence of calcium are shown in Figure 2B–D, respectively. We observed that their K*_d_ value increased in these conditions, revealing the dissociation from ED-LDLR. In order to quantify the effect of these modifications, the ratios between these values and the K*_d_ in normal conditions were calculated (Figure 2E–G), with a high ratio reflecting a high inhibition of the binding in modified conditions. All three clones lost affinity at pH 6.0 characteristic to endosomes, the clones 41 and 47 being mostly affected. These results suggested that peptides could dissociate from LDLR once inside the endosomes, the same as LDL particles. 

However, these clones could bind the ED-LDLR at pH 5.0. According to Huang et al. [34], ED-LDLR seemed to have different conformations at pH 6.0 and 5.0, and we could not exclude the possibility that this modification favors the peptides’ binding. Moreover, because LDL is released in endosomes and that LDLR to not reach physiologically the lysosomes, we hypothesized that LDLR does not present molecular adaptations for an acidic environment, such as pH 5.0.

Moreover, the absence of calcium seemed to decrease the binding of our clones to the target, similar to the natural ligands, suggesting their binding to the same epitope.

These data were then used to calculate the percentage of phage clone dissociation from LDLR at pH 6.0, pH 5.0, and in the absence of Ca^2+^. Considering that K*_d_ was inversely related to the strength of binding (i.e., a low K*_d_ value reflects a strong binding), the apparent affinity constant (K*_a_ = 1/K*_d_) was calculated and assimilated to the maximal binding. Then, the percentage dissociation from the LDLR in these various conditions was expressed as a percentage difference from the maximal binding. These results confirmed that all clones (but mainly clones 41 and 47) dissociated from LDLR at pH 6.0 and in the absence of Ca^2+^ (Figure 2H).

Taken together, the clones 41 and 47 seemed to be most promising because of their interesting characteristics, and their peptides were synthesized. They showed low K*_d_ (7.12 × 10^−11^ and 8.73 × 10^−11^ M, respectively), were relatively stable against natural ligands (mainly the clone 47), whereas acidic pH, as well as the absence of calcium, promoted their dissociation from LDLR.

### 3.2. Analysis of Selected Peptides

The clone 41 carried the peptide LRPep1 (YHFNGCEDPLCR), whereas clone 47 carried the peptide LRPep2 (HPWCCGLRLDLR). Their probability (P) to be expressed in the phage display library was 6.1% and 38.3%, respectively, which could be considered low compared to other identified sequences with P > 90%. This suggested that these peptides were mostly selected due to their affinity to the LDLR and not because of their high frequency in the library.

Their sequences presented some interesting amino acids and shared motifs. They both presented a pair of Cys, supporting the hypothesis that these peptides bind the LDLR by a mechanism that may be similar to that of natural ligands, as already suggested above. However, the spacing by four residues of the Cys pair in LRPep1 should facilitate the disulfide bridge formation with consequences on LDLR binding activity, while the intramolecular disulfide bridge eventually created in LRPep2 by the neighbor Cys residues should likely not interfere with ligand binding. The presence of Pro and Leu also suggested the importance of the tertiary structure in the binding of peptides to the LDLR [37,39]. Moreover, the peptide LRPep2 presented the pattern “Leu-Arg” in two copies that were distant from the Cys pair, whereas the peptide LRPep1 showed this motif separated by a Cys. Interestingly, this pattern was present 32 times in ApoB100 [40] and 6 times in ApoE [41], while Arg residues were involved in the binding of ApoB and ApoE to LDLR, the mutations in these residues being responsible for the loss of affinity [42,43,44]. Interestingly, LDL was positively charged, similar to peptide LRPep2 (isoelectric point [pI] = 10.63), whereas modified LDL through oxidation or acetylation was negatively charged and showed a lower affinity for LDLR [45]. The positive charge of our peptide LRPep2 was thus a supplemental argument for its specific selection during the phage display experiments.

**Figure 3:**
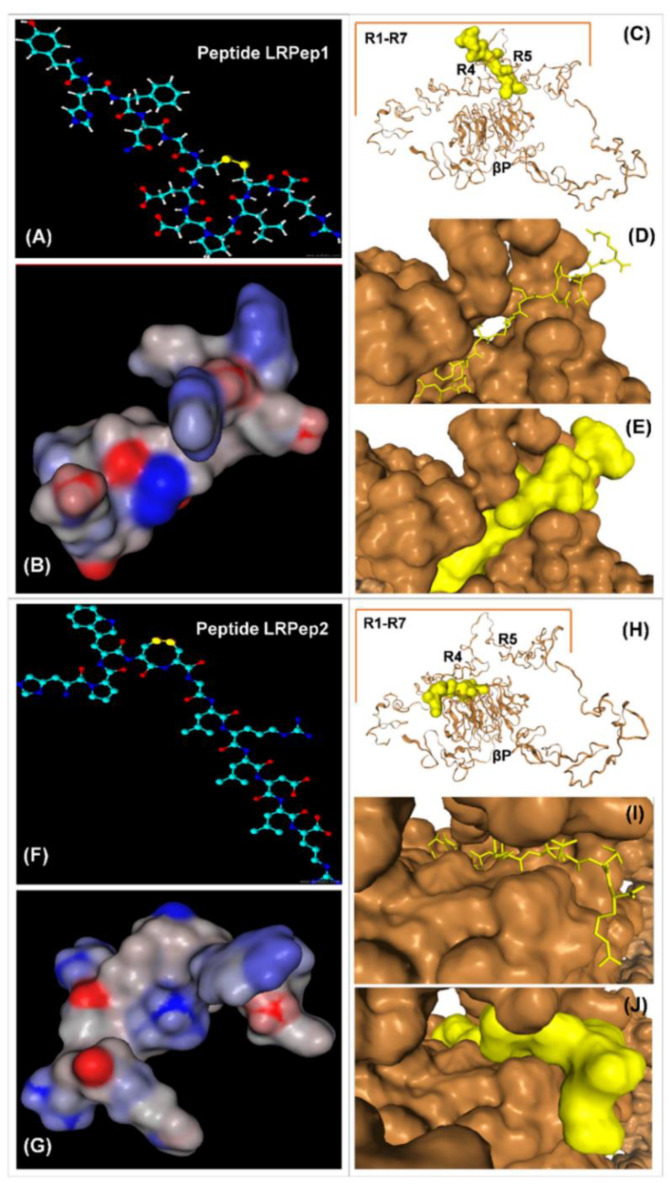
Computational three-dimensional structure (**A**,**F**) and spatial conformation (**B*,*G**) of peptides LRPep1 (**A**,**B**) and LRPep2 (**F**,**G**). The three-dimensional structures of peptides were drawn with ACD/ChemSketch 2.0 software. The peptides are represented with disulfide bridges that could occur in oxidizing conditions between the pairs of Cys (in yellow in **A** and **F**). Their spatial conformations were obtained with MarvinSketch 19.2 software (2019, http://www.chemaxon.com). Interaction of LRPep1 (**C**–**E**) and LRPep2 (**H**–**J**) with ED-LDLR was predicted using the HPEPDOCK program of blind peptide-protein docking (http://huanglab.phys.hust.edu.cn/hpepdock/) [16]. The binding regions of peptides to ED-LDLR are zoomed-in figures **D**–**E** (LRPep1) and **I**–**J** (LRPep2) to better observe the docking models. LRPep1 and LRPep2 appear in yellow, and ED-LDLR in orange. LRPep, LDL receptor-peptide.

The binding of LRPep1 and LRPep2 to ED-LDLR was then investigated using the HPEPDOCK program [22], which allowed the blind docking of peptides to proteins. The results shown in Figure 3 predicted that LRPep1 bound to the linker between R4 and R5 of LDLR, whereas LRPep2 was docked at the interface between R4 and the β-propeller (βP) domain of ED-LDLR, which was much closer to the binding mechanism of LDL [34]. 

At neutral pH, negatively charged residues of R1–R7 (but mainly R4 and R5) in ED-LDLR interacted with positively charged regions in LDL, this interaction being furthermore stabilized by the Trp and His residues in βP domain. At acidic pH, the loss of Ca^2+^ ions (that stabilize the loops in R1–R7 together with disulfide bridges) promoted the LDL release, in addition to the repulsive forces developed by positive charges acquired in these conditions by His^562^ and His^568^ in βP. The interaction of R4 and R5 with βP triggered moreover the allosteric release of LDL [34,43]. As shown above, LRPep1 and mainly LRPep2 dissociated from ED-LDLR at low pH and in the absence of Ca^2+^, pleading for a binding/release from the receptor in a similar manner as the LDL.

### 3.3. In Vitro Evaluation of the Endocytosis Potential of Peptides LRPep1 and LRPep2

**Figure 4:**
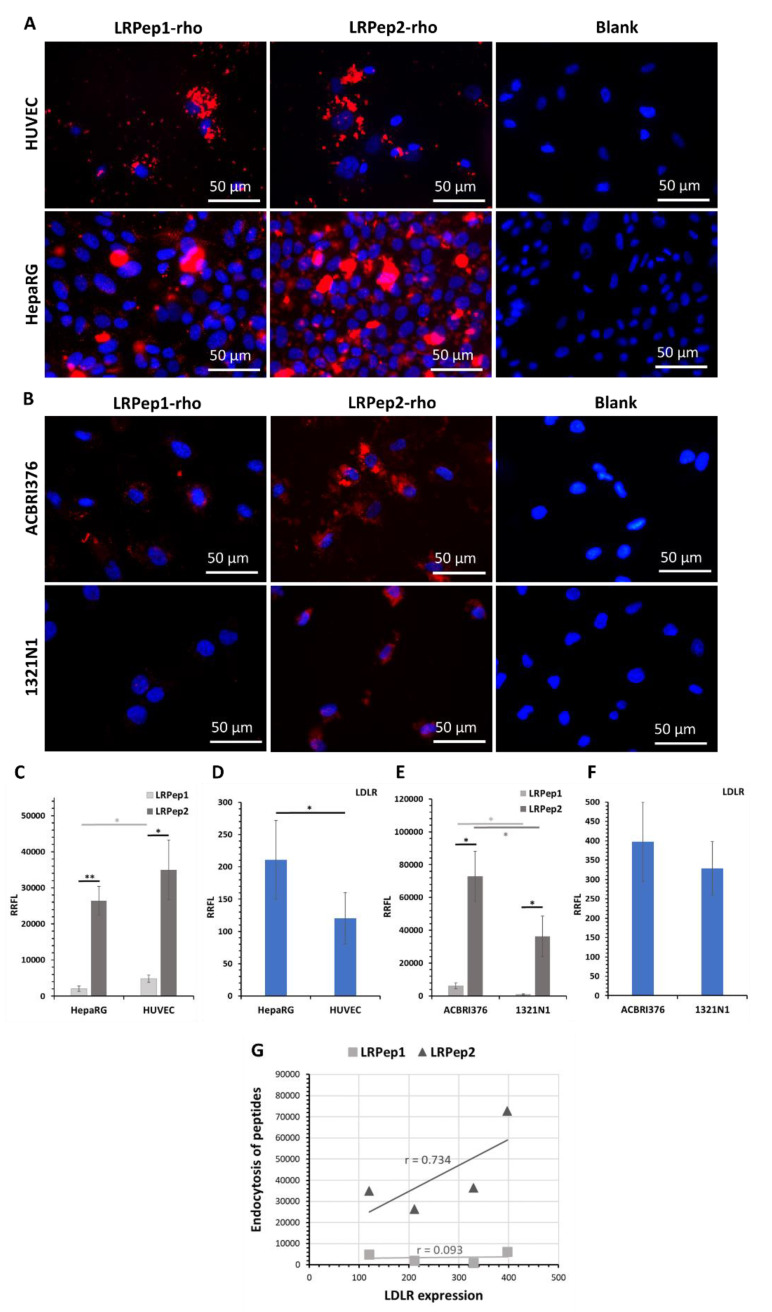
Endocytosis of the peptides LRPep1-rho and LRPep2-rho (stained in red) in (**A**) HUVEC and HepaRG cells and (**B**) ACBRI376 and 1321N1 cells. Nuclei are stained in blue with Hoechst. (**C**–**F**) The endocytosis of peptides, as well as the expression of LDLR, was semi-quantitatively evaluated by the measurement of fluorescent labeling using the ImageJ software and was normalized to the number of cells and to the background, giving the relative ratio of fluorescent labeling (RRFL); *: *p* < 0.05, **: *p* < 0.001. (**G**) The correlation coefficient between the expression of LDLR and the endocytosis of peptides.

In order to evaluate the potential of LDLR-targeted peptides to penetrate cells, the endocytosis of LRPep1-rho and LRPep2-rho was evaluated in the first stage on HUVEC and HepaRG at 200 µM (Figure 4A), and in a second stage on ACBRI376 and 1321N1 at 25 µM (Figure 4B). HUVEC and ACBRI376 are both endothelial cells, the latter being issued from human brain cortex tissue. HepaRG is an in vitro cell model of the liver, playing a crucial role in the clearance of plasma lipids and in which the LDLR is highly expressed. Being a representative of cerebral cells, 1321N1 was employed to observe the possibility of peptide delivery in these cells.

The endocytosis of peptides and the expression of LDLR were semi-quantitatively evaluated by measuring the fluorescent labeling of cells (endocytosis of peptides: Figure 4C,E; LDLR expression: Figure 4D,F, Supplementary Materials Figure S2).

Because the liver is the predominant organ metabolizing the cholesterol in the body, the expression of LDLR in HepaRG was higher than in HUVEC (Figure 4D, *p* < 0.05). In parallel, we observed better endocytosis of both peptides in HUVEC than in HepaRG, as shown by the higher relative ratio of fluorescent labeling (RRFL, Figure 4C, LRPep1: *p* < 0.01, LRPep2: *p* < 0.05). On the other hand, better endocytosis of both peptides was found in ACBRI376 than in 1321N1 cells (Figure 4E, *p* < 0.05), even if the LDLR expression in these cells showed no statistical differences (Figure 4F). However, in all studied cell models, the endocytosis of peptide LRPep2 was more effective than that of peptide LRPep1 (*p* < 0.001 in HepaRG, *p* < 0.05 in HUVEC, ACBRI376, and 1321N1). With the aim to select the most promising peptide, we calculated the correlation coefficients between the endocytosis of peptides and the LDLR expression (Figure 4G), revealing that LRPep2 endocytosis was positively correlated with LDLR expression (r = 0.734) contrariwise to peptide LRPep1 (r = 0.093).

As an important component of the extracellular matrix, collagen allows the attachment of EC and contributes to its polarity [46]. We could thus hypothesize that EC grown on collagen in our experimental conditions would acquire a certain polarity, with the basolateral membrane in contact with the coated coverslip, as shown by other authors in HUVEC, where collagen and β1 integrin have a basal localization [47]. Based on these results, we concluded that peptide LRPep2 had the most promising potential to be used as a vector, and its evaluation had been pursued in additional experiments.

### 3.4. Colocalization of LRPep2 with LDLR on Mouse Brain Slices and Endothelial Cells

**Figure 5:**
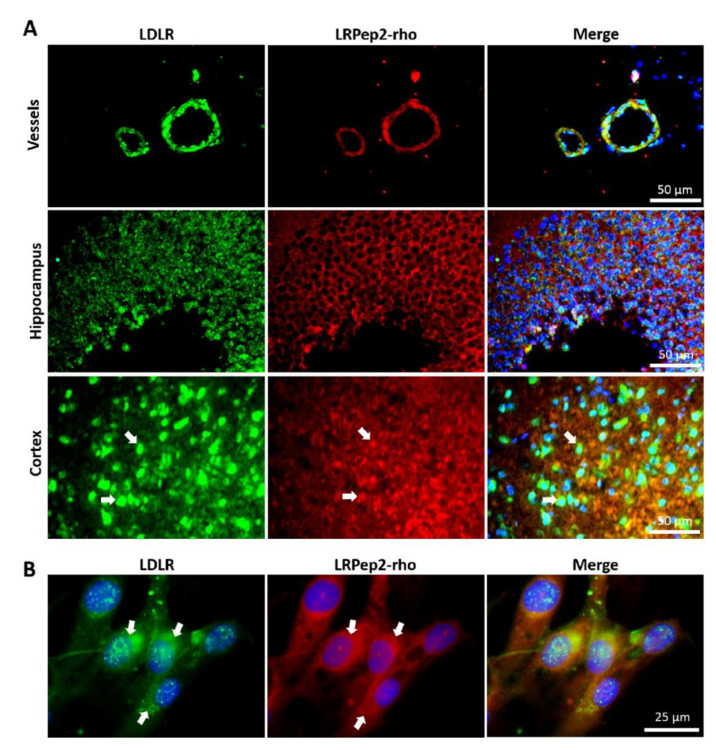
Colocalization of LRPep2-rho with LDLR on mouse brain slices (**A**) and ACBRI376 cells (**B**). LRPep2 appears in red due to the coupled rhodamine, LDLR is stained in green with fluorescein, and nuclei in blue with DAPI. White arrows highlight examples of colocalization areas.

The binding of peptide LRPep2 to LDLR expressed in mouse brain slices (79% sequence identity to human LDLR using BLAST) was verified by IF (Figure 5A), and their colocalization was quantified using the JACoP plugin of the ImageJ software, with the Mander’s coefficient (M) reflecting the percentage of colocalization of the peptide LRPep2 with LDLR [48]. Interestingly, a good colocalization between LRPep2-rho (in red) and LDLR (highlighted by fluorescein in green) was observed at the level of blood vessels (in yellow on merged microphotographs, M = 76.97%). We also observed a large colocalization in the cortex (M = 89.04%, some colocalization areas were highlighted by white arrows) and in a lower proportion in the hippocampus (M = 67.95%). These results suggested the ability of LRPep2 to target LDLR expressed by EC, independently of the blood vessel size, as well as LDLR expressed by cerebral cells, its expression in these different areas being previously observed by IHC (Figure S3). The binding of peptide LRPep2 to the LDLR was moreover confirmed by IF on ACBRI376 cells (Figure 5B), where the peptide LRPep2-rho colocalized with the green staining of LDLR (M = 74.35%), as shown by white arrows in the figure.

The residues 500–550 of the LDLR protein recognized by the anti-LDLR antibody belong to its extracellular domain and are highly conserved in vertebrates, where it does not share sequence homology with other proteins, as revealed by Basic Local Alignment Search Tool (BLAST). This pleads for specific LDLR detection in the studied cell models and consequent specific binding of LRPep2 to ED-LDLR. At this point, we could state that LRPep2 specifically targeted LDLR.

### 3.5. In Vitro Study of the Endocytosis Mechanism of Peptide LRPep2

The endocytosis mechanism of peptide LRPep2 had been studied on the cell models described above and on differentiated N18(H) neuroblastoma cell line by the colocalization of LRPep2-rho with caveolae and lysosomes. The goal of this experiment was to compare the mechanism of endocytosis (i.e., caveolae, lysosomes) borrowed by LRPep2 in various cell types (i.e., EC, astrocytes, neurons, hepatocytes) susceptible to be accessed once the peptide is injected in vivo. These are important aspects for in vivo biodistribution as well as for subsequent bioavailability and metabolization of the vector peptide coupled to the carried pharmacological compound.

**Figure 6:**
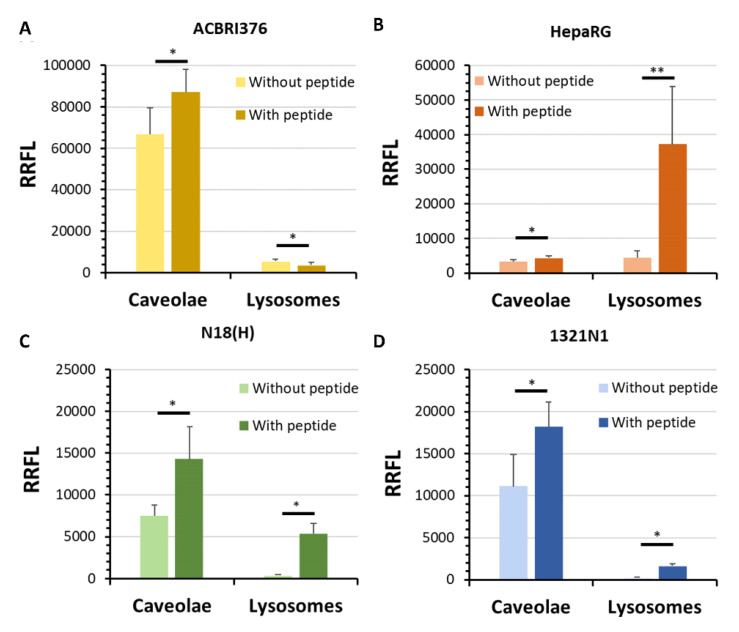
Semi-quantitative analysis of fluorescent labeling of caveolae and lysosomes when ACBRI376 (**A**), HepaRG (**B**), N18(H) (**C**), and 1321N1 (**D**) cells were incubated or not with peptide LRPep2 by the measurement of the RRFL. *: *p* < 0.05, **: *p* < 0.001.

In ACBRI376 cells (Figure 6A), the peptide LRPep2 was endocytosed by the non-degradation pathway involving caveolae, as revealed by the increased fluorescence observed for caveolin-1 (*p* < 0.05), whereas the fluorescence of lysosomes, highlighted by the detection of LAMP1, was decreased in the presence of peptide LRPep2 (*p* < 0.05). Moreover, we observed on microphotographs some large spots of caveolin-1 that colocalized with packs of rhodamine (white arrows on Figure S4, M = 82.7%), suggesting the presence of endocytosis vesicles comprising the peptide LRPep2. The bypass of the lysosome pathway in ACBRI376 was in accordance with previous studies, revealing the localization of LDLR in membrane fraction rich in caveolin [11], where its colocalization with the transferrin receptor was observed [12].

On the contrary, in HepaRG cells, a significant increase of fluorescence was observed for lysosomes (Figure 6B, *p* < 0.001), being in accordance with the involvement of hepatocytes in the metabolism of cholesterol. The role of LDLR in the clearance of plasma lipids in the liver is crucial and, in this organ, the endocytosis of the complex ligand-LDLR will be followed by their dissociation in early endosomes and the hydrolysis of cholesterol in lysosomes, whereas the LDLR is recycled to the plasma membrane [49]. Finally, both pathways seemed to be borrowed by peptide LRPep2 to penetrate in N18(H) and 1321N1 cells (Figure 6C,D, respectively).

Considering that cells were grown in a monolayer but not to confluence, these results could not be interpreted in terms of transcytosis and showed some limitations. Indeed, cells did not form tight junctions sealing EC, and, in these in vitro conditions, we only observed the triggering of endocytosis and hypothesized the pathway followed in different cell types. However, we supported here the possibility that LDLR is an interesting receptor for the specific BBB crossing, as previously suggested by other groups but less explored until now [10,11,12].

Further experiments should state on the in vitro transcytosis ability of LRPep2 in these cells, for example, by cultivating them on transwell inserts until confluence and exposing them to LRPep2. The presence of LRPep2 in the basolateral compartment and its quantification could answer this crucial point.

### 3.6. In Vivo MRI Evaluation of USPIO-LRPep2

**Figure 7:**
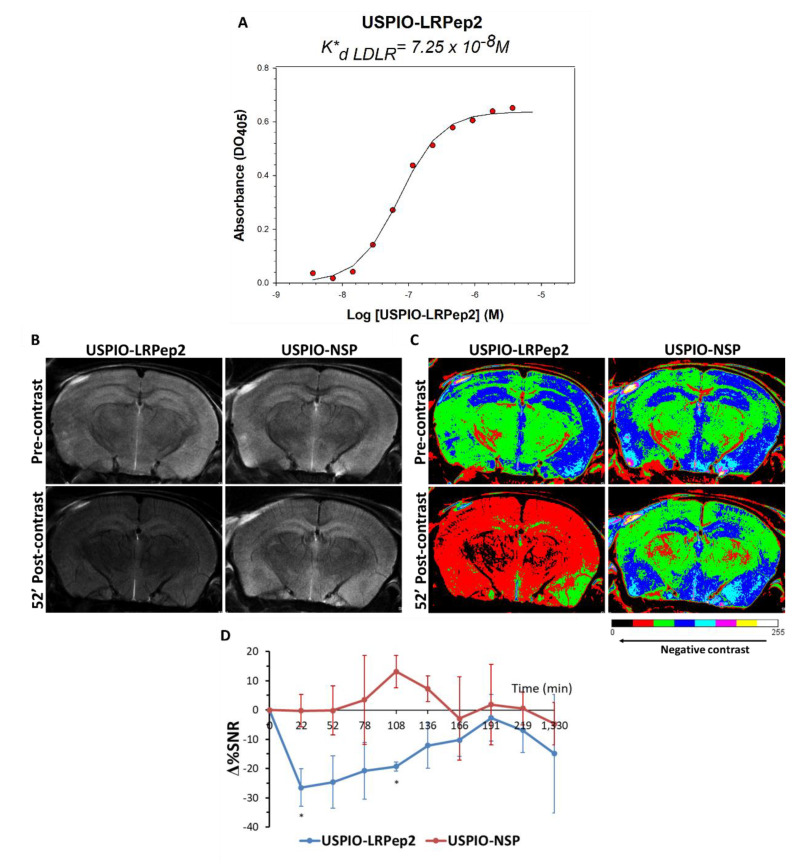
(**A**) The apparent dissociation constant (K*_d_) of ultrasmall superparamagnetic particles of iron oxide (USPIO)-LRPep2 for LDLR. The inflection point of the curve corresponded to the apparent dissociation constant (K*_d_) of USPIO-LRPep2 for the binding to LDLR, and the strength of the affinity was inversely proportional to the K*_d_ value. (**B**–**D**) *In vivo* evaluation of the blood-brain barrier (BBB) crossing ability of USPIO-LRPep2 by MRI. (**B**) Representative raw coronal MRI images of the brains (bregma: −1.64 mm) of NMRI (Naval Medical Research Institute) mice acquired with the rapid acquisition with relaxation enhancement (RARE) protocol (spatial resolution = 48 µm) before (pre-contrast) and 52 min after injection of USPIO derivatives (post-contrast). Accumulation of USPIO derivatives (negative contrast agents) led to the darkening of the brain tissue. (**C**) Color overlay images, allowing to better visualize the negative contrast produced by USPIO, which is directly proportional to the red and black pixels. (**D**) Analysis of the percentage change of signal-to-noise ratio (Δ%SNR) produced by USPIO on MRI images of the brain, measured by the ImageJ software. A decreased Δ%SNR corresponded to the negative contrast generated by USPIO derivatives. *: *p* < 0.05.

The peptide LRPep2 was grafted to USPIO (USPIO-LRPep2) in order to evaluate in vivo its ability to access into the brain. First, the affinity of USPIO-LRPep2 for ED-LDLR was evaluated by determining its K*_d_ by ELISA (Figure 7A), showing a good affinity in the range of nanomolar (K*_d_ = 7.25 × 10^−8^ M) and proving the ability of USPIO-LRPep2 to target LDLR. However, its lower affinity compared to that of LRPep2 displayed by phages (K*_d_ = 8.73 × 10^−11^ M) could be attributed to a different number of peptides exposed by each supramolecular entity, i.e., 1–2 peptides/USPIO and 5 peptides/phage, respectively.

The potential of USPIO-LRPep2 to access the brain was explored by MRI on NMRI mice injected with USPIO-LRPep2 or USPIO-NSP (grafted with a non-specific peptide, used as control), allowing in a first step to observe the presence of USPIO derivatives at the level of mouse brains. A global darkening was observed on 52 min (average timing) post-contrast images of the brain of mice injected with USPIO-LRPep2, whereas mice injected with USPIO-NSP did not present this contrast (Figure 7B, color overlays in Figure 7C). The analysis of this contrast, measured on MRI images and normalized to the noise and to the pre-contrast signal, confirmed these results with the increase of the negative contrast (Figure 7D). A notable negative contrast was present until more than 2 h post-injection for USPIO-LRPep2 (*p* < 0.05 at 22 min and 108 min), whereas no negative contrast was observed for USPIO-NSP. Then, the negative contrast progressively returned to the basal level and disappeared after approximately 3 h. These results suggested the retention of USPIO-LRPep2 at the brain level, and, thanks to the high affinity of LRPep2 to LDLR, we hypothesized that this phenomenon is due to the targeting of this receptor, contrariwise to USPIO-NSP.

### 3.7. In Vivo Biodistribution of USPIO Derivatives

**Figure 8:**
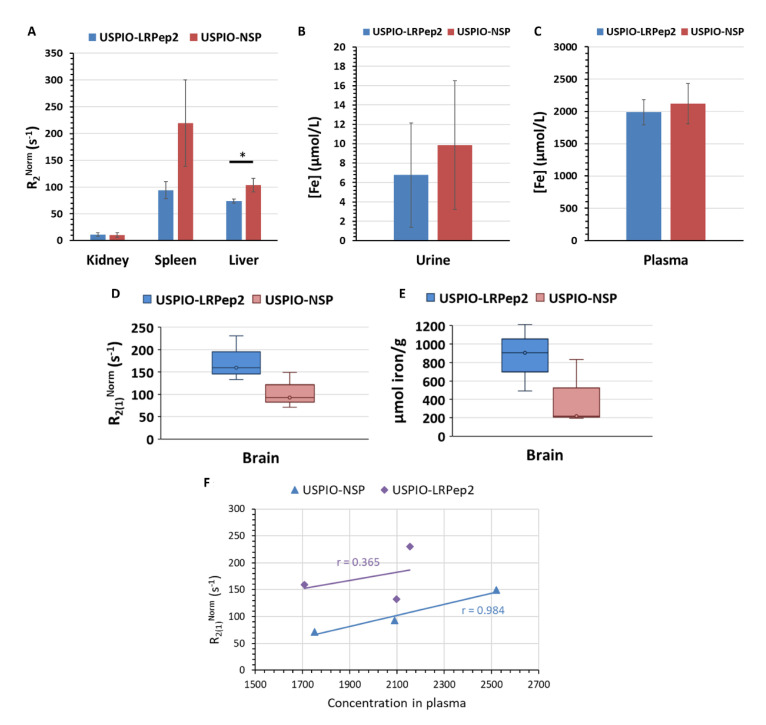
Biodistribution of USPIO-LRPep2 and USPIO-NSP at 55 min post-injection. (**A**) R_2_^Norm^ (=1/T_2_^Sample^ − 1/T_2_^Control^) for each USPIO derivative in the kidney, the spleen, and the liver. * *p* < 0.05. (**B**,**C**) Iron concentration (µmol/L) in urine and plasma, respectively. (**D**) R_2(1)_^Norm^ for each USPIO derivative in the brain. (**E**) Iron concentration (µmol/g of dried tissue) in brains after digestion in acidic conditions. (**F**) Correlation coefficients between the R_2(1)_^Norm^ of USPIO derivatives in the brains and their concentrations in the blood.

The biodistribution of our USPIO derivatives was studied by the measurement of the transversal relaxation times (T_2_) of organs collected at the optimal timing observed by MRI (55 min post-injection). The normalized relaxation rates obtained (R_2_^Norm^ = 1/T_2_^Sample^ − 1/T_2_^control^) are shown in Figure 8. Both USPIO derivatives, but mainly USPIO-NSP, were massively taken up by the spleen and the liver (Figure 8A); these organs containing macrophages are involved in the clearance of many molecules, and especially in iron recycling [50]. A low contribution of kidneys in their elimination was observable, in accordance with the low R_2_ in urine (Figure 8B). This distribution was characteristic of USPIO [32,51]. By contrast, the high plasma concentration of both USPIO derivatives confirmed that they were still circulating in the bloodstream (31% of the injected dose, ID, for USPIO-LRPep2 and 34% of ID for USPIO-NSP, respectively) at this time after injection (Figure 8C).

Concerning the brain, the fast (R_2(1)_^Norm^) component of the relaxation rate [32] obtained after the biexponential fitting of the T_2_ relaxation curve was higher in mice injected with USPIO-LRPep2, suggesting its brain penetration (Figure 8D). The dosage of iron in the brains of injected mice supported these results (Figure 8E); the concentrations being normalized to non-injected mice in order to selectively reflect the iron contained in USPIO. Interestingly, the R_2(1)_^Norm^ of USPIO-LRPep2 seemed to be not related to the plasma concentration of USPIO-LRPep2, as shown by the low correlation coefficient (r = 0.365, Figure 8F), meaning that the free fraction in the bloodstream did not influence this parameter and suggesting the specific presence of USPIO-LRPep2 at the level of brains. On the contrary, USPIO-NSP showed a high positive correlation coefficient between R_2(1)_^Norm^ and its concentration in the plasma (r = 0.984, Figure 8F), meaning that R_2(1)_^Norm^ was directly influenced by the free fraction of USPIO-NSP present in the bloodstream.

Based on the iron concentrations in brains, we calculated the percentage of injected dose per gram of dried tissue (%ID/g) of our USPIO derivatives. Other groups have estimated the %ID/g of different molecules in the brain, ranging between 0.02% and 1% [52,53,54]. Concerning our USPIO derivatives, we obtained higher %ID/g values (USPIO-LRPep2 = 13.5%; USPIO-NSP = 6.6%) that could be attributed to the expression of iron concentration per weight of dried tissue. This could be corrected by assuming a mean brain hydration of 77% (USPIO-LRPep2 = 3.7%; USPIO-NSP = 1.5%). However, it was difficult to compare our results to the other ones because of the different expressions of the initial injected dose, the time point analyzed, the type of the injected agent, and the region of the brain. The ability of nanoparticles to access into the brain was also dependent on the employed coating. Indeed, USPIO injected by Shanehsazzadeh et al. [53], being the most similar contrast agent to our USPIO derivatives, was coated with dextran, whereas we used PEG. Interestingly, even if it is generally admitted that USPIO cannot cross the BBB without functionalization, it is known that PEG facilitates brain penetration [55,56,57], already shown previously by our group [32]. This property could explain the relatively high %ID/g of USPIO-NSP. Nevertheless, the ratio of USPIO-LRPep2/USPIO-NSP for %ID/g revealed that USPIO-LRPep2 was 2 times more concentrated in mouse brains than USPIO-NSP, once more revealing the specific presence of USPIO-LRPep2 in this organ.

### 3.8. Detection of USPIO Derivatives in Mouse Brains by Perls’-DAB Staining

**Figure 9:**
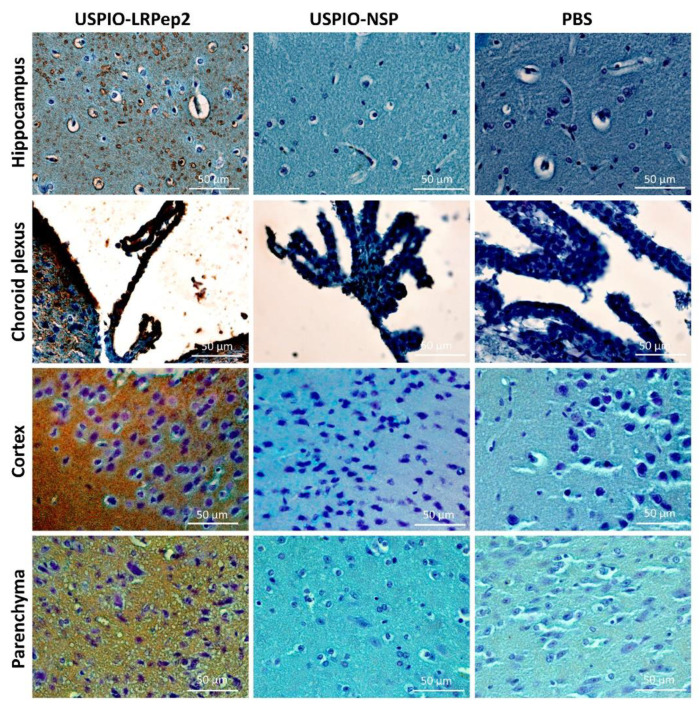
Perls’-DAB brown staining of USPIO derivatives in mouse brains collected at 55 min post-injection. Mice injected with PBS were used as a negative control. USPIO derivatives are stained in brown by the DAB.

USPIO-LRPep2 and USPIO-NSP were detected on mouse brain slices by the Perls’-DAB staining, highlighting the iron present within these nanoparticles, and thus the *in situ* presence of USPIO derivatives (Figure 9). We observed brown staining for mice injected with USPIO-LRPep2 in different areas of brain slices (hippocampus, choroid plexus, cortex, and parenchyma in the hippocampus area), whereas no staining was visible on brain slices of mice injected with USPIO-NSP. This last result, identical to that obtained for mice injected with PBS used as a negative control, suggested that USPIO-NSP quantity that penetrated the brain tissue was inferior to the detection limit of this method. Moreover, the iron detected by NMR, as shown above in Figure 8, could probably be attributed to the residual USPIO-NSP, present in capillaries or the non-specific brain infiltration, as discussed previously; the Perls’-DAB staining revealed that USPIO-NSP did not bind the brain tissue.

### 3.9. In Vivo Fluorescence Evaluation of CF770-LRPep2

**Figure 10:**
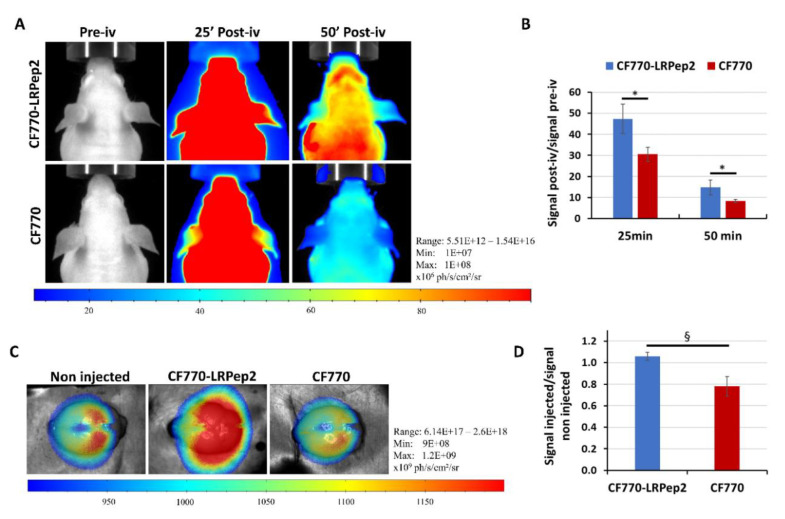
(**A**) FLI images of the brains of nude mice before (pre-iv) and after injection of CF770-LRPep2 or CF770 (post-iv). (**B**) Analysis of the fluorescence observed on FLI images, measured by the M3Vision software in the brain area, and normalized to the pre-iv signal. * *p* < 0.05. (**C**) FLI images of mouse brains ex vivo. (**D**) Analysis of the fluorescence of the brains ex vivo normalized to the brain signal of a non-injected mouse. § *p* = 0.05.

Finally, we performed FLI experiments in order to corroborate the MRI results by a different in vivo imaging method. A higher fluorescence was observed in the area of the brain of mice injected with CF770-LRPep2 as compared to the dye alone (Figure 10A, whole-body images in Figures S5 and S6), and this observation was confirmed by the analysis of the fluorescence (normalized to the signal before the injection) at both studied post-injection times (*p* < 0.05, Figure 10B). After brain collection, the signal of CF770 ex vivo seemed to be identical to that of the non-injected mouse, whereas CF770-LRPep2 allowed us to observe a better fluorescence (Figure 10C). The fluorescence measured on ex vivo brains and normalized to the signal of the non-injected mouse showed a difference (*p* = 0.05) between both compounds (Figure 10D). Moreover, these compounds were massively taken by the liver (CF770-LRPep2) and the kidneys (both CF770 and CF770-LRPep2) (Figures S5 and S6), contrariwise to USPIO derivatives that were taken by the liver and the spleen for iron metabolism.

## 4. Conclusions

The BBB is a real challenge for the development of CNS therapeutic or diagnosis tools, restricting brain availability. LDLR is expressed at low levels in BBB EC, and its role in the brain is poorly elucidated. However, Molino et al. [12] showed that primary rat brain microvascular EC expressed 9-fold higher LDLR levels than the cerebral cortex and that LDLR was expressed essentially at the apical membrane. The authors hypothesized that BBB LDLR could be involved in the transport of other ligands than ApoB-bound cholesterol because of the *in situ* synthesis of cholesterol or that it might be involved in neurologic disorders associated with a compromised brain cholesterol metabolism. In Alzheimer’s disease (AD), some LDLR genetic variants seem to be associated with disease risk or prevention [58] both because LDLR is a receptor of ApoE, which represents a risk factor of AD depending on the expressed allele, and because LDLR has been shown to regulate the brain-to-blood amyloid-beta clearance [59].

In order to facilitate brain access, we identified an LDLR-targeted peptide, called LRPep2, showing promising characteristics as a vector for BBB crossing. The amino acid sequence of this peptide presented patterns known to be involved in the binding of ApoB and ApoE to the LDLR. The disulfide bridge occurring between the vicinal Cys residues in LRPep2 was distantly placed from the two “Leu-Arg” patterns in the peptide sequence, all these amino acid motifs being crucial for the ligand binding to LDLR. Moreover, LRPep2 docking at the interface between R4 and the βP domain of ED-LDLR predicted a binding mechanism similar to that of LDL. It had a good affinity against LDLR, even in the presence of natural ligands, whereas acidic pH and the absence of calcium decreased its binding, suggesting a binding/release from the receptor in a similar manner as the LDL.

LRPep2 was first evaluated in vitro, revealing its endocytosis through a caveolae-dependent pathway in BBB EC, whereas the lysosome pathway was bypassed. The lysosome degradation of pharmacological compounds vectorized by LRPep2 within hepatocytes would promote their inactivation and clearance, preventing, in this way, eventual adverse effects in non-targeted tissues and organs. Additionally, the preferential endocytosis of these compounds through the caveolae pathway in cells of the CNS could facilitate their brain delivery and subsequent activity. Once coupled to USPIO (USPIO-LRPep2), in vivo experiments suggested the potential of our LDLR-targeted peptide to be used as a vector for the BBB crossing, as shown by the presence of nanoparticles in mouse brains by MRI, NMR, and histology, as compared to non-specific nanoparticles. We also tested our LDLR-targeted peptide using FLI after coupling to the fluorescent dye CF770 (CF770-LRPep2), showing a better accumulation of this complex in the brain as compared to the dye alone. Even if we obtained some variability during all in vivo experiments, globally, in vivo results tended to support the ability of our peptide LRPep2 to facilitate the brain penetration compared to the NSP. Moreover, the variability seemed to be more important for USPIO-NSP, probably due to the grafted peptide.

Altogether, our study showed that the LDLR is an interesting target for the crossing of the BBB and that our peptide LRPep2 is a promising vector to promote brain penetration through LDLR. In the future, the coupling of LRPep2 to a therapeutic or diagnostic agent would be useful to show the facilitation of brain access.

## 5. Patents

The peptides described in this article are the subject of a patent deposited by the University of Mons.

## Figures and Tables

**Table 1:** Amino acid sequences obtained from the 29 selected clones and their probability to be expressed in the phage display library (P). Consensus motifs are underlined, whereas Cys pairs are shown in bold.

Clones	Sequences	P (k > 0) (%)
1	GHIPTCLTPMCR	40.9
9	HSIRDGFRSTPV	99.9
10	TGQTVTGLSYIF	99.4
16	KVVSLSALQSMT	100
21	WTSQPHLQHVDD	91.5
17, 23	KVWSLVNQGGQF	39.3
2, 7, 8, 12, 14, 18, 22, 24, 35, 36, 44, 48	AHLPTSMLKGQG	99.9
38	GHLAVNMPRASL	100
40	HHTGCLSPLSCS	99.9
34, 41	YHFNGCEDPLCR	6.1
42	HWKVTTWNSSTV	89.8
46	SGVYKVAYDWQH	33.9
47	HPWCCGLRLDLR	38.3

**Table 2:** Apparent dissociation constant **(**K*_d_) of selected clones, half-maximal inhibitory concentration (IC_50_) of apolipoproteins B (ApoB) and E (ApoE), and ratios IC_50_/K*_d_, reflecting the inhibitory effects of ApoB and ApoE on the clones’ binding. The IC_50_/K*_d_ value was directly proportional to the strength of the clones’ binding to the extracellular domain of LDLR (ED-LDLR).

Clones	K*_d_	IC_50_ ApoB	IC_50_ ApoE	Ratio ApoB	Ratio ApoE
1	1.36 × 10^−10^	6.23 × 10^−19^	1.67 × 10^−20^	4.55 × 10^−9^	1.22 × 10^−10^
36	2.23 × 10^−11^	8.31 × 10^−19^	1.71 × 10^−8^	3.71 × 10^−8^	763.97
38	2.41 × 10^−10^	3.49 × 10^−9^	2.57 × 10^−9^	14.48	10.66
40	2.17 × 10^−12^	2.95 × 10^−9^	3.33 × 10^−9^	1361.51	1536.90
41	7.12 × 10^−11^	2.32 × 10^−11^	1.90 × 10^−10^	0.33	2.67
47	8.73 × 10^−11^	3.54 × 10^−9^	1.26 × 10^−7^	40.54	1443.01

## Supplementary Materials

The following are available online at https://www.mdpi.com/2079-7737/9/7/161/s1, Figure S1: Individual affinities of the 50 clones isolated from the 3rd round of panning evaluated against the ED-LDLR and the BSA, Figure S2: Fluorescent immunostaining of LDLR in HUVEC, HepaRG, ACBRI376, and 1321N1 cells, Figure S3: Detection of LDLR on mouse brain slices by immunohistochemistry, Figure S4: Colocalization of LRPep2-rho with caveolae and lysosomes in ACBRI376 human brain microvascular EC, Figure S5: Whole-body FLI images of 3 mice before (pre-iv) and after CF770 injection (50 min post-iv), Figure S6: Whole-body FLI images of 4 mice before (pre-iv) and after CF770-LRPep2 injection (50 min post-iv). Methods: protocols for biopanning, clones’ isolation and amplification, evaluation of the binding to ED-LDLR of isolated clones by ELISA, evaluation of the inhibitory 50% (IC_50_) of ApoB and ApoE in competition with LDLR-targeted clones, evaluation of the influence of pH and calcium on the binding of LDLR-targeted clones, and DNA purification and sequencing of the selected clones.
