# Supplementary material for: Development of an LDL Receptor-Targeted Peptide Susceptible to Facilitate the Brain Access of Diagnostic or Therapeutic Agents
Source: Biology (Basel). 2020 Jul 11;9(7):161. doi: 10.3390/biology9070161 (PMC7407834; doi:10.3390/biology9070161)
Supplement: Supplementary file 1 [file biology-09-00161-s001.zip › Supplementary Materials_Methods.pdf]

Supplementary Materials - Methods

Development of an LDL receptor-targeted peptide susceptible to facilitate the brain access of diagnostic or therapeutic agents

Séverine André [1], Lionel Larbanoix [2], Sébastien Verteneuil [1,a], Dimitri Stanicki [1], Denis Nonclercq [3], Luce Vander Elst [1], Sophie Laurent [1,2], Robert N. Muller [1,2] and Carmen Burtea [1,*]

[1] Department of General, Organic and Biomedical Chemistry, NMR and Molecular Imaging Laboratory, University of Mons, Avenue Maistriau 19, Mendeleïev Building, B-7000 Mons, Belgium; Severine.ANDRE@umons.ac.be; Sebastien.Verteneuil@uliege.be; Dimitri.STANICKI@umons.ac.be; Luce.VANDERELST@umons.ac.be; Sophie.LAURENT@umons.ac.be; Robert.MULLER@umons.ac.be; Carmen.BURTEA@umons.ac.be

[2] Center for Microscopy and Molecular Imaging, rue Adrienne Bolland 8, B-6041 Gosselies, Belgium; Lionel.LARBANOIX@umons.ac.be

[3] Department of Histology, University of Mons, Pentagon – 1B, Avenue du Champ de Mars 6, B-7000 Mons, Belgium; Denis.NONCLERCQ@umons.ac.be

[a] Present address: Developmental Neurobiology Unit, GIGA Neurosciences, University of Liège, C.H.U. B36, avenue Hippocrate 15, B-4000 Liège, Belgium

* Correspondence: Carmen.BURTEA@umons.ac.be; Tel.: +32–65373814

Supplementary Materials - Methods

1. *Biopanning*

The phage displayed random library of linear dodecapeptides (Ph.D.-12, New England Biolabs Inc., Bioké, Leiden, The Netherlands) was screened against the extracellular domain (ED) of LDLR (ED-LDLR, Recombinant Human LDLR, R&D Systems, Abingdon, Oxon, UK). The target was immobilized in a well of a 96-well plate at a concentration of 25µg/ml in $NaHCO_3$ 0.1M, pH 8.6 (immobilization buffer). After overnight incubation at 4°C and elimination of the target solution, this well and another one, used for negative selection, were blocked for 2 hours at 4°C using immobilization buffer completed with 0.5% of Bovine Serum Albumin (BSA). Wells were emptied and rinsed 6 times with TBSC-T (Tris-HCl 50mM, NaCl 150mM, $CaCl_2$ 2mM, pH 7.4, completed with Tween-20 at the concentration of the round, see below). Non-specific phages were removed by the library pre-incubation ($2x10^{11}$ phages in 200µl of TBSC-T) with the BSA-coated well for 60 minutes before the transfer of the solution in the ED-LDLR-coated well for 120 minutes. In order to enhance the selection pressure, the conditions of incubation were modified gradually at each panning round: (i) Tween-20 concentration was increased from 0.1% for the 1st round to 0.3% for the 2nd round and 0.5% for the 3rd round; (ii) the incubation times of the library were increased for the BSA-coated well (90 and 120 minutes respectively) and decreased for the ED-LDLR-coated well (90 and 60 minutes respectively).

The phages-containing solution was discarded, and the well was rinsed 10 times with TBSC-T. Phages bound to the target were eluted by addition of glycine-HCl buffer 0.2 M (pH 2.2) completed with 0.1% BSA and stirring for 20 minutes. The output was recovered in a tube and the acidic solution was neutralized by Tris-HCl buffer 1M ($C_4H_{11}NO_3 \cdot HCl$ 1M, pH 9.1).

After each biopanning, phages were amplified in *E. coli* ER2738 (New England Biolabs). Phage Outputs were added to a bacteria culture diluted 1:100 from an overnight culture (LB 25g/L, Luria Bertani Broth Miller, Sigma-Aldrich, Bornem, Belgium; completed with 40µg/mL of tetracyclin [stock prepared at 200 mg in 50 mL of ethanol 50%]). After 4.5 hours of stirring (160 rpm) at 37°C, the culture was centrifugated (15 minutes, 10000 rpm, 4°C) and the supernatant was added to Polyethylen Glycol 8000 20% - NaCl 2.5 M (1/6 of the supernatant volume) to precipitate phages overnight at 4°C. The solution was centrifugated (20 minutes, 10000 rpm, 4°C) allowing to obtain a pellet containing phages, this one being resuspended in 1mL of TBS (Tris-HCl 50mM, NaCl 150mM, pH 7.5) and transferred in a tube before a new centrifugation (5 minutes, 7000 rpm, 4°C). The supernatant was added to 1/6 of PEG-NaCl and incubated on ice for one hour. Once more, the solution was centrifugated (10 minutes, 14000 rpm, 4°C) before resuspending the pellet in 20-200µL of TBS (depending on the pellet size). A final centrifugation (1 minute) allowed to obtain the amplified Output in the supernatant. This one was used for the subsequent biopanning.

2. *Clones isolation and amplification*

The third Output (not amplified) was used for the isolation of 50 individual clones. For this purpose, a culture of *E. coli* at mid-log (optical density read at 600nm [OD_{600}] around 0.5) was incubated for 5 minutes with dilutions of the output (10^1 to 10^5 in LB). Then, the solutions were transferred to agarose (25g/mL LB, $MgCl_2 \cdot 6H_2O$ 5mM, 7g/L agarose) maintained at 45°C, quickly mixed and spread on LB/agar/IPTG/XGal petri dishes (1L of LB supplemented with 15g of agar and 1mL of IPTG/XGal solution [1.25g isopropyl-beta-d-thiogalactoside IPTG, 1g 5-bromo-4-chloro-3-indolyl-beta-d-galactopyranoside XGal, 25mL dimethylformamide DMF]). After overnight incubation (upside down, maximum 18 hours), 50 isolated blue colonies were collected and amplified in microculture plates. Each colony was placed in one well containing 1.5 mL of *E. coli* overnight culture diluted 1:100 in LB and plates were stirred for 4.5 hours at 37°C (600 rpm). Plates were centrifugated (20 minutes, 3000 rpm, 4°C) and the supernatant of each well was transferred in tubes containing PEG-NaCl for overnight precipitation. After centrifugation (10 minutes, 14000 rpm, 4°C), the pellets were resuspended in TBS/glycerol (1:1) for long storage at -20°C.

The phage titer is determined by absorbance measurement and calculated by this equation:

$$\text{Phage concentration (particles/mL)} = \frac{(OD_{269} - OD_{320}) \text{ x } 6\text{x}10^{16} \text{ x } 50}{9312} \quad (1)$$

In this equation, OD_{269} represents maximal absorption of phage DNA, OD_{320} reflects a potential contamination, $6\text{x}10^{16}$ corresponds to the number of particles/mL at OD_{269}=1, as recommended by the Georges Smith lab, 50 is the dilution made of the phage solution in TBS, and 9312 corresponds to the number of bases per viral particle. In order to calculate the K^*_d of a phage clone, the phage concentration per liter was converted in moles per liter by using the number of Avogadro.

3. Evaluation of the binding to ED-LDLR of isolated phage clones by ELISA

The selection of the hits among the isolated clones was performed by the evaluation of their binding to the target (ED-LDLR) as compared to the non-specific binding to BSA. The target was immobilized at 10µg/mL in immobilization buffer (one well per clone + one blank) and wells were blocked (all LDLR-coated wells and an equal number of empty wells) as previously described. After each incubation step, the wells were rinsed 3 times with TBSC-T (0.5% Tween-20) using an automatic microplate washer (Beckman Coulter, Analis, Suarlée, Belgium).

Each clone was then incubated with LDLR and BSA-coated wells at a concentration of $5\text{x}10^{11}$ phages in 100µl of buffer for 2 hours under stirring (350 rpm). The blank was incubated with the buffer. Then the HRP-conjugated monoclonal anti-M13 antibody (Amersham Pharmacia Biotech Benelux, Roosendaal, The Netherlands) was incubated for 1 hour (dilution 1:5000 in TBSC with 0.5% BSA) to detect bound phages. The revelation was performed by the addition of ABTS-H_2O_2 0.05% solution (22mg 2,2'-azino-bis[3-ethylbenzothiazoline-6-sulphonic acid diammonium salt], ABTS, in 100mL sodium citrate 50mM pH 4). The OD_{405} (differential filter: 630nm) was measured using a microplate reader (StatFax-2100, Awareness Technology, Fisher Bioblock Scientific, Tournai, Belgium).

The same protocol was used for the evaluation of the apparent dissociation constant (K^*_d) using a range of 10 dilutions (1:2) of selected clones, starting at $2 \text{ x } 10^{12}$ phages.

4. Evaluation of the inhibitory concentrations 50% (IC_{50}) of ApoB and ApoE in competition with LDLR-targeted clones

The IC_{50} of natural ligands (ApoB and ApoE, Abcam, Cambridge, UK) was determined using a constant concentration of phages (corresponding to the K^*_d, prepared at $2\text{x}K^*_d$ in TBSC-Tween 0.5%) and a range of 8 dilutions (1:4) of competitors starting at 5 nM in TBSC complemented with 0.1% BSA and 0.5% Tween-20.

LDLR-coated wells were pre-incubated with 50 µL of competitors for 30 minutes (ambient temperature, 350 rpm) before adding 50 µL of phages in each well and incubating for 1.5 hour. Blanks were incubated with TBSC-T whereas the non-inhibited binding was determined by the incubation of the target with phages (pre-incubation with TBSC-T). The detection of bound phages was performed as previously described.

5. Evaluation of the influence of pH and calcium on the binding of LDLR-targeted clones

The protocols were adapted from Huang et al. Concerning the influence of pH, after the blocking step, LDLR coated wells were incubated with TBSC 3 x 10 minutes before the incubation with dilutions of phages (range of the K^*_d) for 2 hours (ambient temperature, 350 rpm). After discarding the phage solutions and rinsing the plate, wells were incubated with TBSC (pH 5 or 6, supplemented with 0.5% BSA) for 30 minutes under stirring [33]. The detection of bound phages was performed as previously described.

Concerning the influence of calcium, the rinsing buffer was TBSM-T (Tris-HCl 20mM, NaCl 100mM, pH 8, supplemented with 0.5% Tween-20) free of calcium. Phages were diluted (range of the

K^{*}_{d}) in TBSM supplemented with EDTA 20mM and 1% BSA (TBSM-EDTA-BSA). After the blocking step, wells were incubated 3 x 10 minutes with TBSM-EDTA-BSA before the incubation with phages (2 hours, ambient temperature, 350rpm). The anti-M13 antibody was prepared in TBS completed with 0.5% BSA [33].

6. DNA purification and sequencing of the selected clones

The DNA of each selected clone was extracted and purified using the phenol/chloroform method. The sequencing was performed (Beckman Coulter Genomics, Grenoble, France) using a 20-base primer (5′- CCC TCA TAG TTA GCG TAA CG –3′) complementary to a sequence located 96 bp downstream of the inserted sequence. DNA sequences were analyzed to identify the sequence coding for the inserted peptide located between the pIII leader sequence and the N-terminus of the mature pIII protein of M13 bacteriophage (flanking sequences: CACTCT – X – GGTGGAGGTTCG) before translating it into the coded amino acid sequence (JaMBW 1.1 software, http://bioinformatics.org/JaMBW).

The probability of expression of one specific sequence is calculated using the frequencies of each amino acid in the library, these frequencies being given by the manufacturer. For a given sequence, the frequencies of each amino acid are multiplied to obtain the value p reflecting the probability of expression of this sequence. This value p is then multiplied by the complexity n of the library, in our case $n = 4.1 \times 10^{15}$ different peptides, giving us the number λ of independent clones expressing this specific sequence. The probability $P(k)$ that the employed library comprises exactly this k clones expressing this sequence is calculated by the Poisson distribution:

$$P(k) = e^{-\lambda}.\lambda^{k}/k! \; [k=0, P(0) = e^{-np}] \tag{2}$$

Finally, the probability P(k>0) that the library comprises at least one clone expressing the given sequence is calculated by the equation:

$$P(k>0) = 1-e^{-np} \tag{3}$$

© 2020 by the authors. Submitted for possible open access publication under the terms and conditions of the Creative Commons Attribution (CC BY) license (http://creativecommons.org/licenses/by/4.0/).
