# Supplementary material for: Development of an LDL Receptor-Targeted Peptide Susceptible to Facilitate the Brain Access of Diagnostic or Therapeutic Agents
Source: Biology (Basel). 2020 Jul 11;9(7):161. doi: 10.3390/biology9070161 (PMC7407834; doi:10.3390/biology9070161)
Supplement: Supplementary file 1 [file biology-09-00161-s001.zip › Supplementary Materials_Figures.pdf]

Supplementary Materials - Figures

Development of an LDL receptor-targeted peptide susceptible to facilitate the brain access of diagnostic or therapeutic agents

Séverine André [1], Lionel Larbanoix [2], Sébastien Verteneuil [1,a], Dimitri Stanicki [1], Denis Nonclercq [3], Luce Vander Elst [1], Sophie Laurent [1,2], Robert N. Muller [1,2] and Carmen Burtea [1,*]

[1] Department of General, Organic and Biomedical Chemistry, NMR and Molecular Imaging Laboratory, University of Mons, Avenue Maistriau 19, Mendeleïev Building, B-7000 Mons, Belgium; Severine.ANDRE@umons.ac.be; Sebastien.Verteneuil@uliege.be; Dimitri.STANICKI@umons.ac.be; Luce.VANDERELST@umons.ac.be; Sophie.LAURENT@umons.ac.be; Robert.MULLER@umons.ac.be; Carmen.BURTEA@umons.ac.be

[2] Center for Microscopy and Molecular Imaging, rue Adrienne Bolland 8, B-6041 Gosselies, Belgium; Lionel.LARBANOIX@umons.ac.be

[3] Department of Histology, University of Mons, Pentagon – 1B, Avenue du Champ de Mars 6, B-7000 Mons, Belgium; Denis.NONCLERCQ@umons.ac.be

[a] Present address: Developmental Neurobiology Unit, GIGA Neurosciences, University of Liège, C.H.U. B36, avenue Hippocrate 15, B-4000 Liège, Belgium

* Correspondence: Carmen.BURTEA@umons.ac.be; Tel.: +32–65373814

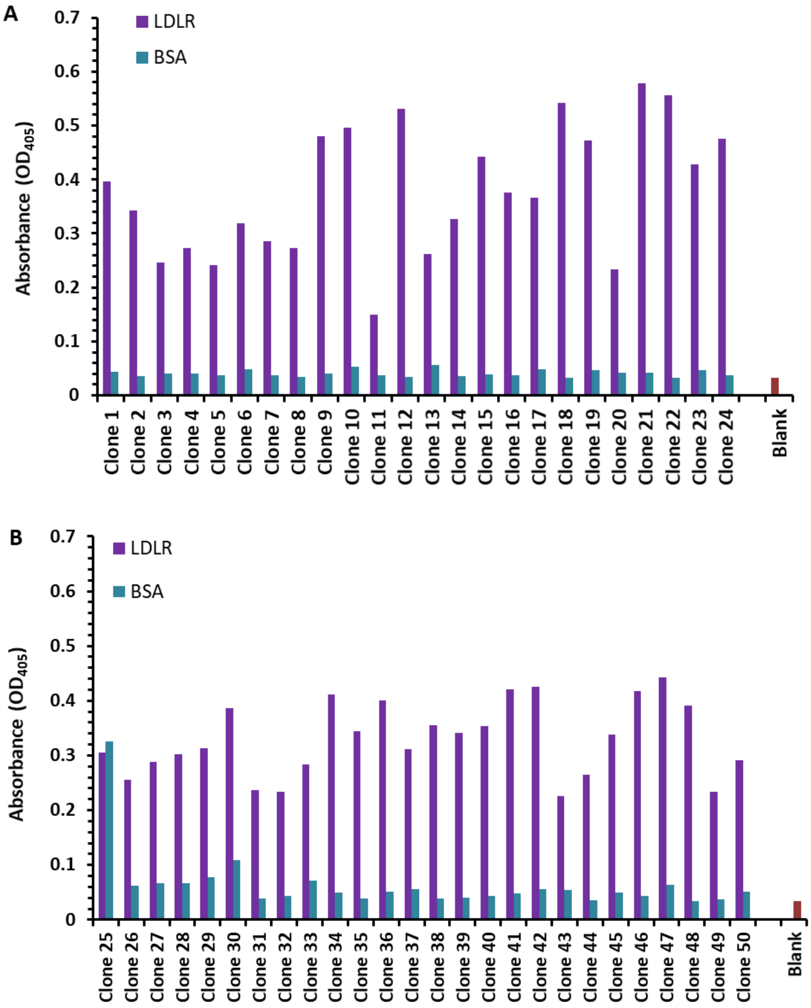

Supplementary Figure S1. Individual affinities of the 50 clones isolated from the 3rd round of panning evaluated against the ED-LDLR and the BSA. (**A**) Clones 1 to 24. (**B**) Clones 25 to 50. The blank corresponds to the non-specific binding of anti-M13 antibody to the ELISA plate.

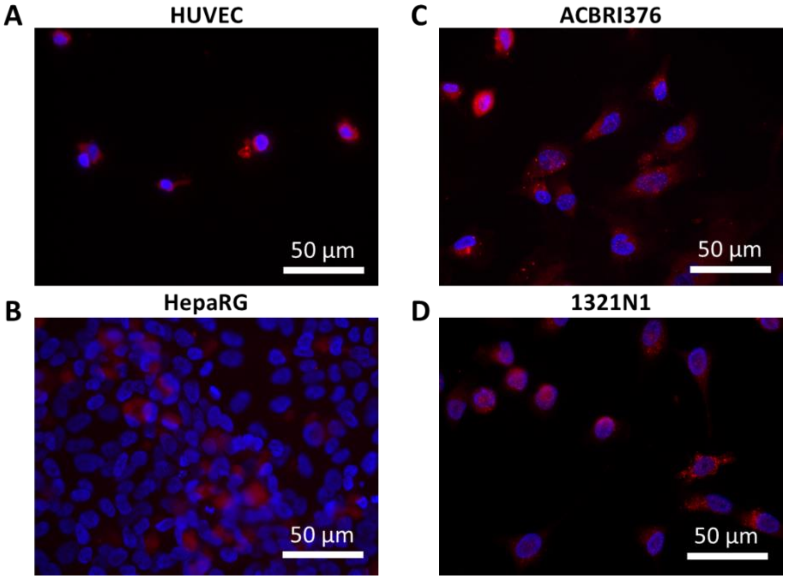

Supplementary Figure S2. Fluorescent immunostaining of LDLR (stained in red with Texas Red) in (**A**) HUVEC, (**B**) HepaRG, (**C**) ACBRI376 and (**D**) 1321N1 cells. Nuclei are stained in blue with DAPI.

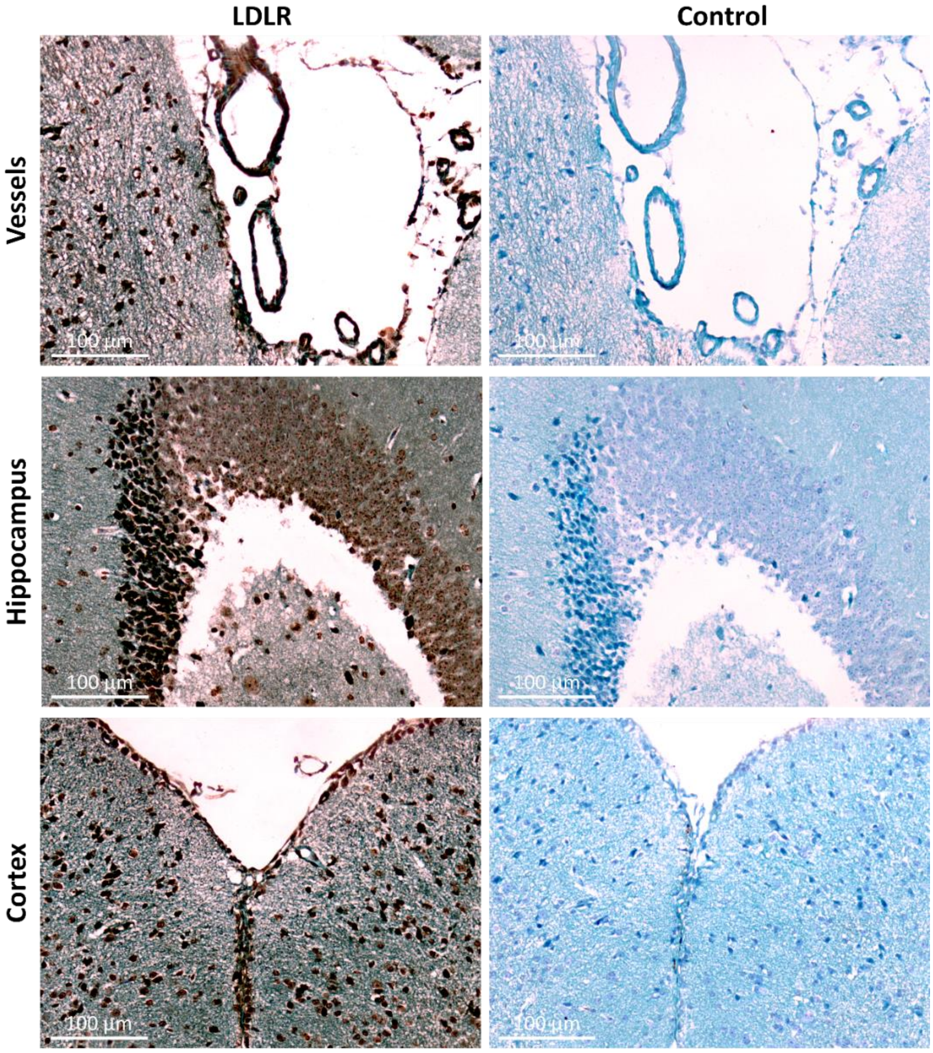

Supplementary Figure S3. Detection of LDLR on mouse brain slices by immunohistochemistry. LDLR is stained in brown by DAB.

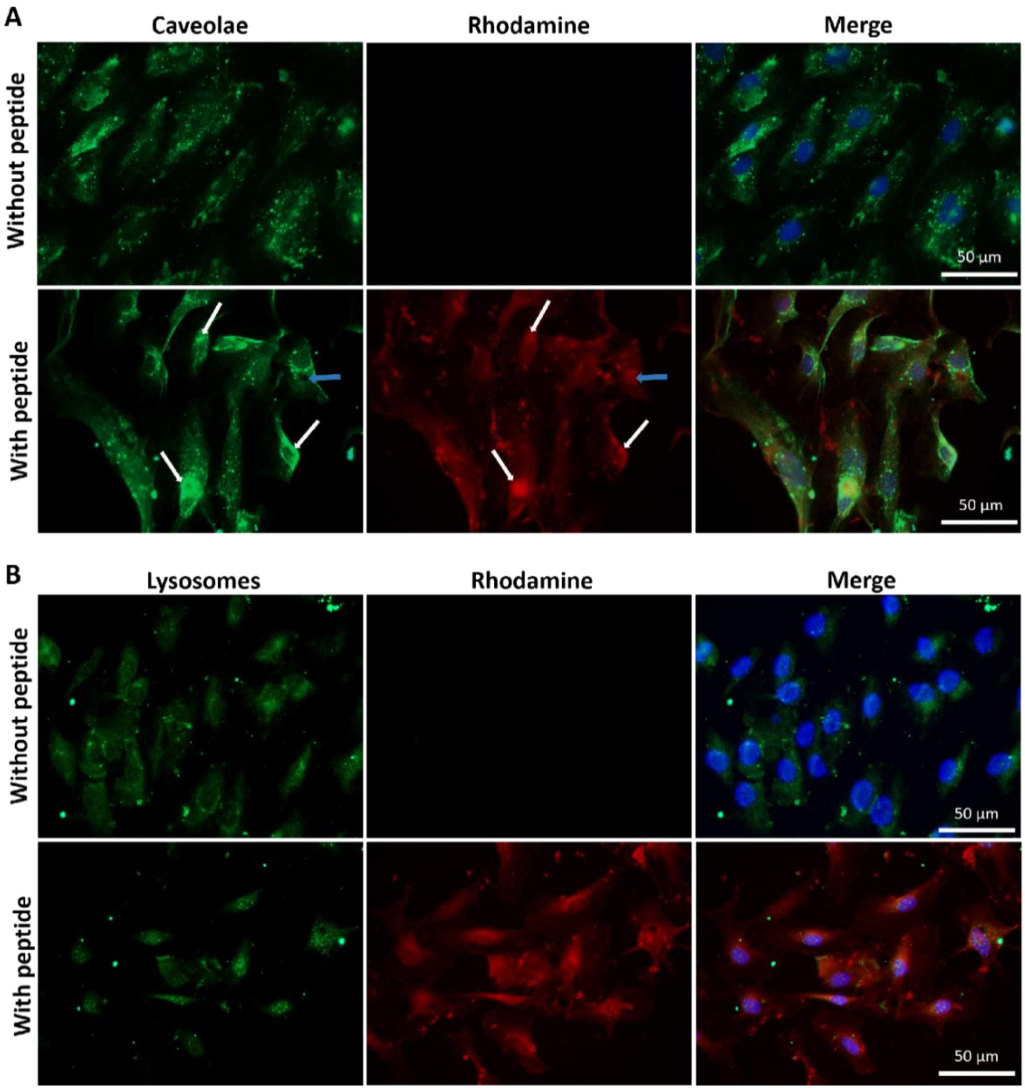

Supplementary Figure S4. Colocalization of LRPep2-rho with caveolae (**A**) and lysosomes (**B**) in ACBRI376 human brain microvascular EC. LRPep2 is stained in red due to its rhodamine, caveolae and lysosomes are stained in green with fluorescein and nuclei in blue with DAPI. White arrows show accumulation of LRPep2-rho that colocalize with large spots of caveolae, but not with lysosomes.

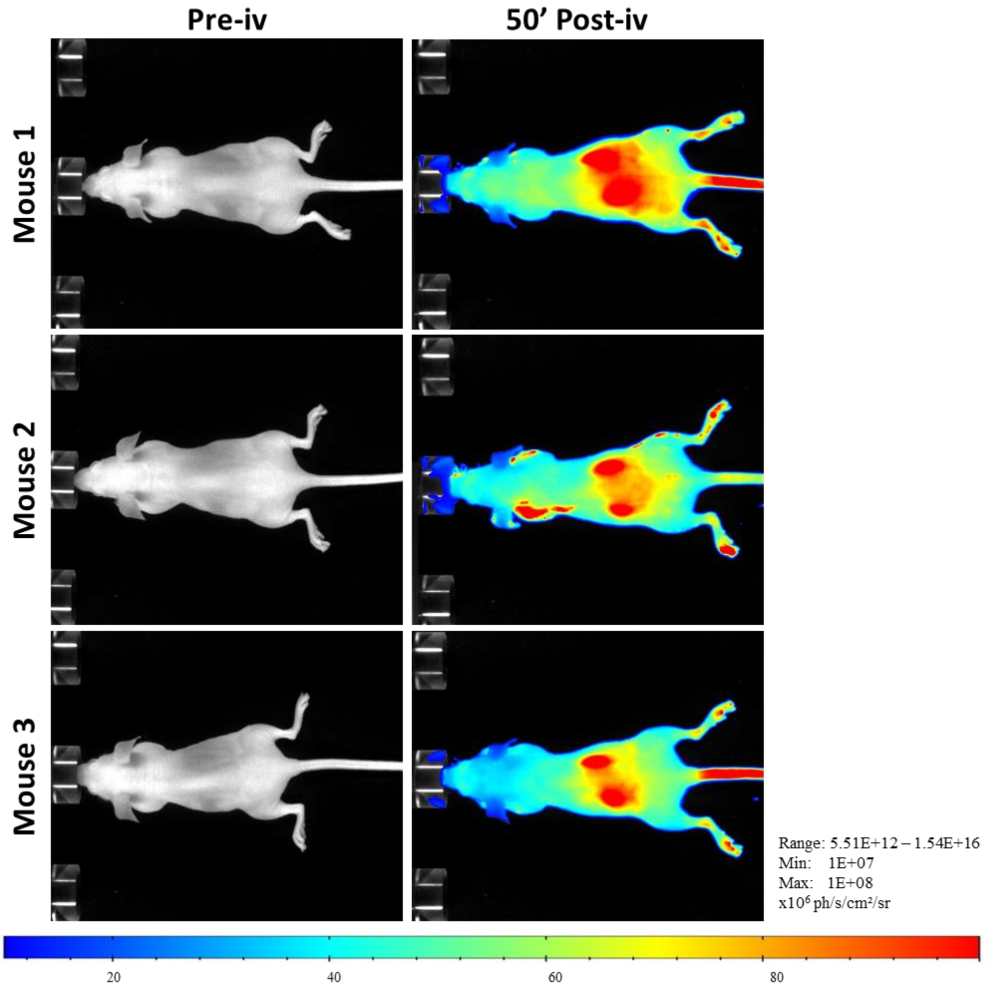

Supplementary Figure S5. Whole body FLI images of 3 mice before (pre-iv) and after CF770 injection (50 min post-iv).

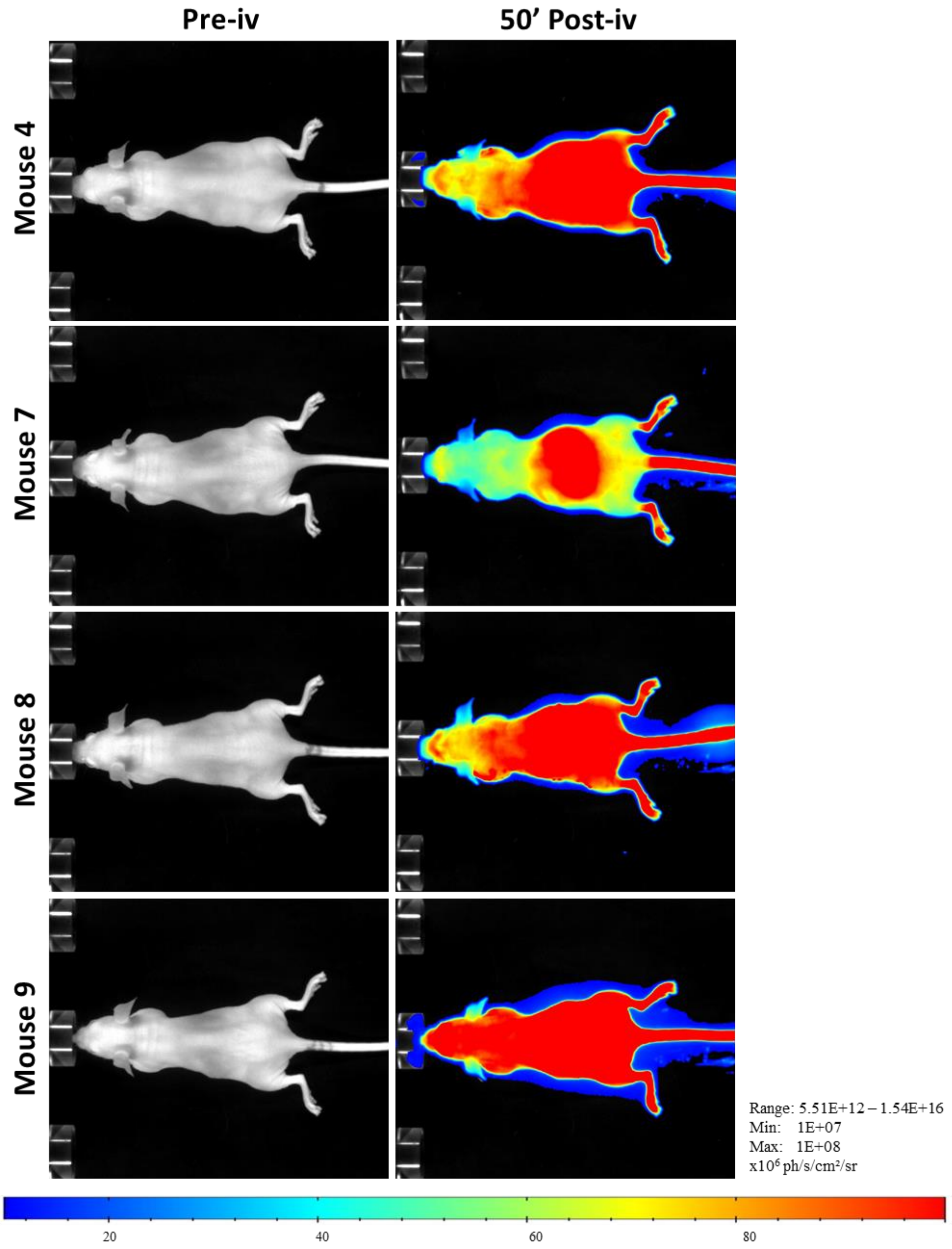

Supplementary Figure S6. Whole body FLI images of 4 mice before (pre-iv) and after CF770-LRPep2 injection (50 min post-iv).

© 2020 by the authors. Submitted for possible open access publication under the terms and conditions of the Creative Commons Attribution (CC BY) license (http://creativecommons.org/licenses/by/4.0/).
